# Machine learning approaches for influenza A virus risk assessment identifies predictive correlates using ferret model in vivo data

**DOI:** 10.1038/s42003-024-06629-0

**Published:** 2024-08-01

**Authors:** Troy J. Kieran, Xiangjie Sun, Taronna R. Maines, Jessica A. Belser

**Affiliations:** Influenza Division, Centers for Disease Control and Prevention, Atlanta, GA USA

**Keywords:** Influenza virus, Machine learning

## Abstract

In vivo assessments of influenza A virus (IAV) pathogenicity and transmissibility in ferrets represent a crucial component of many pandemic risk assessment rubrics, but few systematic efforts to identify which data from in vivo experimentation are most useful for predicting pathogenesis and transmission outcomes have been conducted. To this aim, we aggregated viral and molecular data from 125 contemporary IAV (H1, H2, H3, H5, H7, and H9 subtypes) evaluated in ferrets under a consistent protocol. Three overarching predictive classification outcomes (lethality, morbidity, transmissibility) were constructed using machine learning (ML) techniques, employing datasets emphasizing virological and clinical parameters from inoculated ferrets, limited to viral sequence-based information, or combining both data types. Among 11 different ML algorithms tested and assessed, gradient boosting machines and random forest algorithms yielded the highest performance, with models for lethality and transmission consistently better performing than models predicting morbidity. Comparisons of feature selection among models was performed, and highest performing models were validated with results from external risk assessment studies. Our findings show that ML algorithms can be used to summarize complex in vivo experimental work into succinct summaries that inform and enhance risk assessment criteria for pandemic preparedness that take in vivo data into account.

## Abstract (web summary)

Use of machine learning to summarize complex in vivo data and provide predictive utility for Influenza A virus ferret lethality, morbidity, and transmission models.

## Introduction

Machine learning (ML), a subset of artificial intelligence, has attracted significant interest for its transformative potential across industries, including infectious disease risk assessment^1,2^. By analyzing vast amounts of data and identifying complex patterns, ML algorithms can provide valuable insights and predictions across biological and microbiological research^3,4^. These algorithms allow computers to learn and make predictions from data, enhancing performance by recognizing patterns and relationships, which can be particularly valuable in tasks such as disease diagnosis and risk assessment through the analysis of large datasets^5^. In the case of influenza A viruses (IAV), ML algorithms can predict host susceptibility and transmission dynamics by considering factors such as genetics, host characteristics, and environmental parameters^6,7^, enabling the identification of critical determinants that influence host-virus interactions. However, while selected studies have trained and tested models to predict outcomes of in vivo experimentation with IAV^8–11^, few efforts have been conducted to date that formally include data from in vivo experimentation in model training datasets^12^, and only infrequently have included viral titer-based or clinical parameters from experimentally inoculated animals^13^.

The CDC and WHO have established public health risk assessment tools which aid pandemic preparedness efforts, and are informed in part by data generated in vivo^14,15^. These multi-attribute, additive rubrics provide quantitative approaches to assess the relative impact and emergence of newly identified IAV, to inform resource allocation, development of candidate vaccine viruses, and other public health decisions. However, these tools are not predictive. There is a need for studies to identify not only viral properties and molecular determinants which contribute to key infection outcomes (notably disease severity and transmissibility)^16–18^, but to further assess the relative ability of quantifiable datapoints to predict these outcomes on a more rapid timeframe after viral isolation. While ML algorithms have been employed in the context of IAV risk assessment activities, including in healthcare settings^19^, poultry farms^20^, and surveillance of wild bird populations^21^, studies specifically employing both in vivo and viral metadata which formally contribute to risk assessment rubrics evaluating the emergence and spread of pre-pandemic IAV have not been conducted to date.

Ferrets are frequently employed to inform IAV pandemic risk assessment rubrics as, unlike other small mammalian models, this species permits the coincident study of IAV pathogenesis and transmission^22^. Many laboratories worldwide employ ferrets to study these multifactorial traits, but heterogeneity in experimental designs and other confounders limits the capacity to perform meta-analyses from data collected across different institutions^23,24^. Furthermore, the relatively small sample size of ferret experiments (e.g., *n* = 3 or 4 transmission pairs per virus tested) precludes robust statistical analyses from most studies in isolation^25,26^. For these reasons, despite the critical role played by this species, there have been comparatively few studies performed that examine trends in aggregated in vivo-generated data^16–18,27–29^; these analyses have been generally limited to statistical assessments and linear models, with few predictive modeling studies performed.

To this aim, we aggregated ferret pathogenesis and transmission data collected for risk assessment purposes over a 25-year span, to evaluate which ML algorithms are best suited for in vivo-generated data, and which phenotypic outcomes from in vivo experimentation are most accurately predicted via ML. Once high-performing classification models for lethality and transmission outcomes were established, we tested these models with externally generated risk assessment data from the published literature to validate classification outcomes. Collectively, our findings support that ML algorithms can permit identification of features generated from in vivo experimentation which have high predictive value and can ascertain which molecular features most faithfully predict mammalian phenotypic outcomes in the absence of in vivo experimentation.

## Results

### Source data and supervised classification models tested

**Fig. 1:**
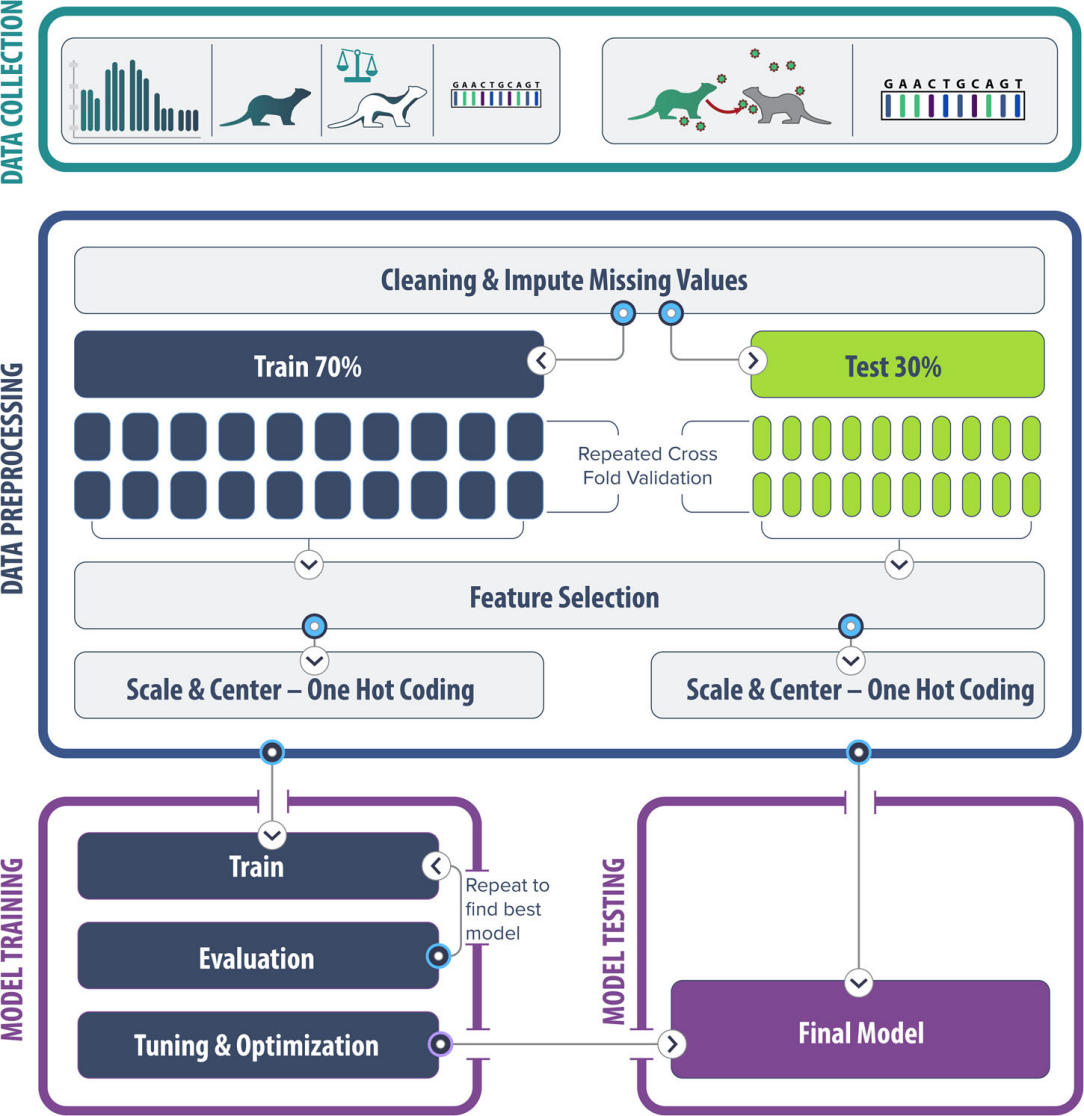
Analysis workflow for generation of models employing machine learning algorithms. IAV metadata and results from in vivo experimentation are collected from pathogenicity (to inform lethality and morbidity classifications, top left depiction) and respiratory droplet transmissibility (to inform transmission classification, top right depiction) experiments in ferrets. Extensive data preprocessing was conducted to train numerous supervised classification models (encapsulating 11 different ML algorithms) and assess relative model performance. Final model selection led to additional model training and tuning, with all chosen models tested with both internally generated data and several external datasets for validation purposes. Illustrations in this figure were generated by the US Centers for Disease Control and Prevention.

Ferrets (*n* = 717) were inoculated with an extensive panel of 125 unique influenza A viruses (Supplemental Table 1) by standard high-dose intranasal instillation, with multiple virological and clinical parameters captured post-inoculation as specified in the methods. Informed by these data, three overarching supervised classification models (lethality, morbidity, and transmissibility) were developed to classify and predict, on a per-ferret basis, a binary outcome (yes/no for lethality, yes/no for high weight loss, ≥50%/<50% for transmission in a respiratory droplet setting) from aggregated ferret data (Fig. 1). Each classification model was trained on three different source data types emulative of information generated during standard risk assessment activities (see Table 1 for key features included in models and rationale for inclusion). The standard data type included viral titer and clinical symptoms obtained from virus-inoculated ferrets, sequence-predicted receptor-binding preference and polymerase activity, with limited other metadata. The molecular only data type included no data from virus-inoculated ferrets and was informed solely by sequence-based and other viral metadata available prior to in vivo experimentation. A combined data type pooled all available data parameters from both standard and molecular data sets. Details of all 9 models evaluated are presented in Table 2.

**Table 1:** Summary of key features used to train classification models on different outcome variables

Variable name	Variable description	Rationale for inclusion	Phenotypic implications
lethal (outcome variable)	Binary ferret survival (yes/no) over 14 days p.i.	Lethality models trained on ability to predict known mortality outcomes of ferrets inoculated with different IAV	Lethal outcome in ferrets serves as a parameter for predicting disease severity in humans^14^
wt_loss_high (outcome variable)	Binary ferret weight loss ≥14.5% over 14 days p.i.	Morbidity models trained on ability to predict high weight loss outcomes of ferrets inoculated with different IAV	IAV with a capacity to cause substantial weight loss in ferrets have a higher potential to cause more serious illness in humans^42^
RD_trans_ext (outcome variable)	Binary ferret transmission (yes/no) as defined by both infectious virus detection in NW specimens and seroconversion of contact animals	Transmission models trained on ability to predict successful IAV infection of contact animals in a respiratory droplet transmission setting	Novel and emerging IAV with airborne transmissible phenotypes are considered a higher pandemic risk^23^
MBAA^*a*^	HPAI viruses that also possess a multibasic amino acid (MBAA) cleavage site	MBAA cleavage site represents a known correlate of IAV virulence in mammals	IAV with a MBAA cleavage site often cause more severe disease in mammals than those lacking this feature^74^
RBS^*a*^	Predicted HA receptor binding preference	Enhanced binding to α2,6 linked sialic acids represents a known correlate of IAV transmissibility in mammals	Capacity of IAV to bind to α2-6 sialic acid receptors present in mammalian upper respiratory tract is linked with higher transmissibility^40^
PA^a^	Predicted polymerase activity	Human-like polymerase activity is associated with enhanced virus replication in mammalian cells and cold sensitivity	IAV with high polymerase activity in mammalian cells can support virus replication in the cooler temperatures of mammalian nasal cavity^46^
Origin_orig/Origin^a^	Origin_orig refers to the host species of isolation, with the exception of human isolates resulting from spillover infection of avian or swine viruses, which are categorized as avian origin or variants, respectively. Origin is a binary grouping of Origin_orig (avian or mammalian (non-avian))	Depending on the species of isolation, IAV may have distinct molecular characteristics to support virus replication or adaptation in mammals, which can contribute to varied phenotypic outcomes	IAV isolated from different host origins frequently possess unique genetic characteristics that support virus replication and transmission within these hosts; cross-species IAV infection often requires acquisition of specific adaptive changes in viral genomes^46^
wt_loss	Peak maximum weight loss (reported as percentage of pre-inoculation weight) over 14 days p.i.	Weight loss from preinoculation baselines during IAV infection represents a primary measurement of disease severity in ferrets	Weight loss ≥25% from preinoculation baselines is often employed as a humane endpoint^42^
temp/temp_5	Peak rise in pre-inoculation temperature (in degrees C) over 14 days p.i. (temp) or over first 5 days p.i. only (temp_5)	Elevated body temperature from preinoculation baselines during IAV infection represents a primary measurement of disease severity in ferrets	Pyrexia can be detected following infection with both seasonal and zoonotic-origin IAV in both humans and ferrets^42^
AUC	Area under the curve (AUC) of ferret NW specimens titered over the first 4 (AUC_4) or 6 (AUC_6) days p.i.	AUC gated over different day spans can vary by virus^17^	Higher AUC values can be indicative of higher viral titers and/or longer duration of viral shedding over a defined period of time
slope_1,3_	Measurement of virus growth or decay in NW specimens between days 1 and 3 p.i.	Infection progression parameters represent an emerging metric to assess IAV replication in the mammalian URT in ferrets^58^	Titers of well-adapted mammalian-origin IAV in NW typically peak early p.i., leading to negative slope_1,3_ values, with the converse detected for zoonotic-origin IAV^75^
peak_inoc/d3_inoc	Peak NW titer over days 1–6 p.i. (peak_inoc) or day 3 p.i. only (d3_inoc)	Infectious IAV titer in NW specimens represents a standard metric to assess replication in the mammalian URT in ferrets	Higher viral loads in NW specimens can indicate more robust replication in mammalian upper respiratory tract
HA/Subtype	IAV HA subtype only (HA) or HA and NA subtypes reported together (Subtype)	IAV subtype represents standard information captured and contextualized during risk assessment activities	HA and NA subtypes harbor key information regarding viral antigenic, genetic, and structural characteristics, in addition to indicating host immunological status against such viruses^46^
All Positions	HA 17, 18, 21, 83, 110, 126, 128, 137, 138, 143, 155, 148, 160, 186, 187, 190, 192, 193, 196, 197, 214, 225, 226, 227, 228, 239, 255, 318, 329, 387, 443, 446, 496; PB2 271, 590/591, 627, 701; RBS; PA	Amino acid residues in the HA and PB2 proteins with known or potential roles in viral virulence, transmissibility, or host adaptation	Amino acids at these residues are associated with virulence and transmission modulation in mammalian hosts; some of these have been identified in human isolates from zoonotic viral infections^39^

^a^See Supplemental Table 1 for strain-specific designations for MBAA, RBS, PA, and Origin.

**Table 2:** Description of supervised classification models trained and tested in this study

Model name^a^	Classification^b^	Data Type^c^	Algorithm^d^	# Viruses^e^	# Obs yes/no (total)^f^	Important Features^g^
L1	Lethality	Standard	gbm	125	102/615 (717)	wt_loss, MBAA, AUC_6
L1M	Lethality	Combined	gbm	119*	102/615 (717)	wt_loss, MBAA, HA-160T
LM	Lethality	Molecular	gbm	119*	102/615 (717)	HA-214V, HA-160T, HA-496R
M1	Morbidity	Standard	Stacked	125	176/539 (715)	AUC_6, temp_5, MBAA
M1M	Morbidity	Combined	gbm	119*	176/539 (715)	AUC_6, temp_5, MBAA
MM	Morbidity	Molecular	gbm	119*	176/539 (715)	HA-227S, PB2-271T,HA-228G
T1	Transmission	Standard	rf	96	213/262 (475)	AUC_6, slope_1,3_, HA-H5
T1M	Transmission	Combined	rf	94*	213/262 (475)	PB2-627E, HA-21S, HA-138A
TM	Transmission	Molecular	rf	94*	213/262 (475)	PB2-627E, HA-138A, HA-21S
L1-H1N1	Lethality	Standard	gbm	2	3/85 (88)	NA
L1-sim	Lethality	Standard	gbm	18^	900/2000 (2900)	NA
LM-pub	Lethality	Molecular	gbm	78	47/388 (425)	NA
M1-H1N1	Morbidity	Standard	Stacked	2	21/67 (88)	NA
M1-sim	Morbidity	Standard	Stacked	18^	847/2053 (2900)	NA
TM-pub	Transmission	Molecular	rf	33	96/100 (196)	NA

^a^Abbreviation of model evaluated in this study. All models were trained with internally generated data and tested with either internally-generated data (no qualifier), data published from a ferret transmission standardization exercise (H1N1), data simulated from internally-generated data (sim), or data aggregated from previously published literature (pub) (see methods). All features of each model are stated in Supplementary Data 1 and Supplemental Fig. 1.

^b^Classification was lethality (ferret surviving the 14-day p.i. observation period or not), morbidity (ferret lost >14.5% preinoculation body weight over 14 p.i. observation period or not), or transmission (ferret likely to transmit virus ≥50% of the time to a contact animal in a RDT setting or not).

^c^Data type represents the scope of input data used to train or validate each model: standard (inclusive of in vivo-generated data and selected viral molecular information), molecular (inclusive of viral molecular inputs with no in vivo-generated data) or combined (all in vivo and molecular inputs).

^d^ML algorithm governing the model: gradient boosting (gbm), random forest (rf), or ensemble of multiple models (Stacked, see methods).

^e^Number of unique wild-type IAV in the source dataset for testing or training (*, not including dummy variable viruses; ^, proxy number of viruses based on unique combination of HA, RBS, and PA).

^f^Per-ferret yes/no observations based on classification model tested.

^g^Top three ranked for each model trained and tested with internally generated data. NA, models tested with external datasets.

### Iterative testing of disparate machine learning algorithms

**Fig. 2:**
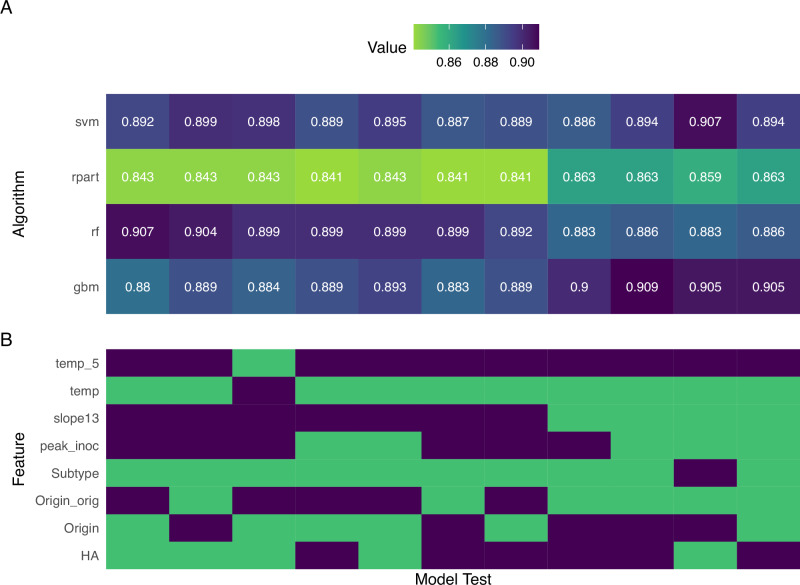
Comparison of lethality standard model balanced accuracy and feature selection iterations. **A** Heat map depicting balanced accuracy performance metrics of four ML algorithms (support vector machine (svm), decision trees (rpart), random forest (rf), and gradient boosting (gbm)) for the lethality standard (L1) model employing different feature selection. Values range from 0 (worst, green) to 1 (best, purple). **B** Feature inclusion for ML algorithms shown. Purple, feature inclusion; green, feature exclusion. All L1 models include AUC_6, MBAA, RBS, and PA features (not shown). Origin_orig: Virus host origin (human, variant, avian, swine, canine), based on lineage (not species of isolation). Origin: Binary virus host origin (avian or mammalian, see methods for definition). Temp: peak rise in pre-inoculation temperature (in degrees C) over 14 days p.i. (temp) or over first 5 days p.i. only (temp_5). slope_1,3_: measurement of virus growth or decay in NW specimens between days 1 and 3 p.i. peak_inoc: peak NW titer over days 1–6 p.i. HA: IAV HA subtype only. Subtype: IAV HA and NA subtypes combined. Feature definitions are also shown and provided in Supplemental Fig. 1 and Supplementary Data 1. Full scope of all model metrics and feature selections for models described in Table 2 are reported in Supplementary Data 2–7.

ML algorithms can vary in the relative weight they give different parameters, leading to variability in outcomes and overall performance metrics. Due to a paucity of previously assessed models employing in vivo data in the context of viral infection, and a systemic lack of head-to-head comparisons of ML performance metrics when employing virological data, we chose to test a panel of 11 different ML algorithms spanning several different ML families (see methods) against all 9 model iterations described above. For each of the 11 ML algorithms tested, multiple iterations of feature selection were tested (Supplementary Data 1), with final model selection informed by assessing 14 performance metrics (area under the curve [AUC], accuracy, balanced accuracy, detection rate, F1, kappa, negative prediction value, positive prediction value, precision, recall, sensitivity, specificity, logarithmic loss, precision-recall AUC), with a focus on balanced accuracy, sensitivity, specificity, and F1 score. A full scope of all metrics calculated, for each feature iteration, for each model, are presented in Supplementary Data 2–7. An example is provided in Fig. 2 showing one performance metric (balanced accuracy) for 4 of the 11 ML algorithms across each iteration of features tested (Supplementary Data 1) for the L1 model (lethality standard). Employing these metrics for all 9 models evaluated, we found a range of variability between the ML algorithm employed and selected features informing each model. However, these assessments identified a consistent trend of top performing (gbm, nnet, rf, ranger) and low performing (glm, rpart) algorithms independent of the outcome metric assessed. Based on these metrics, a final algorithm of gradient boosting (gbm) was selected for lethality and morbidity models, while random forest (rf) was selected for transmission models. The top three features for each final model are presented in Table 2 and discussed in more detail below. Subsequent refinement of all classification models was performed with hyperparameter tuning (Supplementary Data 8) using the sample metrics for finalized model selection.

### Assessments of final model performance and comparison

**Fig. 3:**
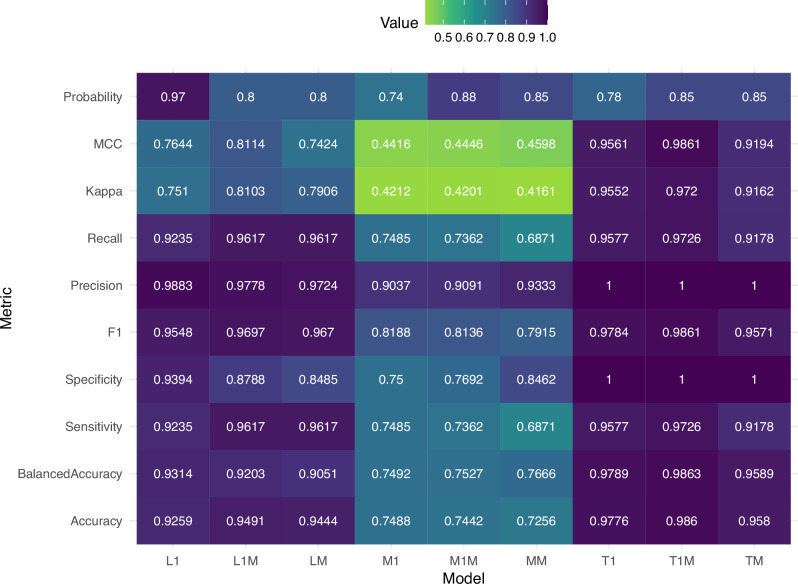
Performance metrics for lethality, morbidity, and transmission classification models. Heat map depicting 9 performance metrics and the probability of prediction threshold determined for 9 models including standard data (L1, M1, T1), molecular only (LM, MM, TM), and combined data types (L1M, M1M, T1M). All 9 model iterations are presented in Table 2. Values range from -1 (no agreement) to 1 (agreement) (MCC, Kappa) or 0 (worst, green) to 1 (best, purple) (all other metrics).

Once final algorithm selection was determined, we next examined in depth the relative performance of all 9 models (lethality, morbidity, and transmissibility, with standard, molecular or combined data types), with a focus on balanced accuracy, sensitivity, specificity, and F1 score metrics. Balanced accuracy was >0.9 among lethality classification models employing a tuned gradient boosting ML algorithm, with the standard (L1) model consistently showing the highest balanced accuracy (0.9314) followed closely by the combined (L1M) and molecular (LM) models (Fig. 3, Supplementary Data 2, 3), demonstrating that all models independent of training data type could accurately categorize both positive (no lethality) and negative (yes lethality) cases among our internal data test sets. Sensitivity values were also >0.92 across all lethality models, emphasizing model competence in correctly recognizing true positive outcomes. Specificity values exhibited greater variability between lethality models but still demonstrated the ability of all lethality models to accurately classify negative events with values of 0.9394 (L1), 0.8788 (LM1), and 0.8485 (LM). The F1 score, a metric that balances recall and precision, was >0.95 across all models, further supporting that all lethality models could correctly balance detection of positive events with a reduction in false positives independent of the data type employed for training.

While the lethality models were found to be generally robust, models assessing morbidity (as measured by maximum weight loss of virus-inoculated ferrets) underperformed. For morbidity, the standard (M1) model was evaluated using the random forest algorithm of a stack of the top individually performing models (neural net, ranger, and gradient boosting), while molecular (MM) and combined (M1M) models employed a tuned gradient boosting algorithm (Supplementary Data 8). Balanced accuracy was consistently very similar across all three morbidity models (0.7492-0.7666) (Fig. 3, Supplementary Data 4, 5). Specificity and sensitivity values were consistent and balanced for M1 (0.75, 0.7485) and M1M (0.7692, 0.7362) respectively, while model MM had more specificity (0.8462) at the cost of sensitivity (0.6871), resulting in consistent balanced accuracy as shown above. The F1 score followed a similar pattern with values ranging from 0.7915 (MM) to 0.8188 (M1). While the stacked model algorithms were the best performing, improvement over the top individually performing algorithms for morbidity was negligible.

For virus transmission by respiratory droplets, standard (T1), molecular (TM), and combined (T1M) models were finalized using a tuned random forest algorithm (Supplementary Data 8), but several others (such as ranger, gradient boosting, and neural net) were comparable competitors. Similar to lethality classification models, all transmission models were very predictive when tested with internally generated data, with balanced accuracy >0.95 for all three models (Fig. 3, Supplementary Data 6, 7). All models possessed maximum specificity, with very high sensitivity values of 0.9726 (T1M), 0.9577 (T1), and 0.9178 (TM). This pattern was similar, with maximum precision and high recall values, resulting in consistently high F1 scores >0.95 independent of the data type employed for model training.

To further compare relative model performance, we employed Matthew’s Correlation Coefficient (MCC), a metric that considers true negatives and positives, and false negatives and positives, producing a high score if only good predictive rates are found for each category. The MCC score ranges from −1 (complete misclassification) to 1 (perfect classification), with zero values being random classification. In agreement with other performance metrics discussed above, MCC supported that transmission classification models were the most accurate, followed by lethality, then morbidity models which performed comparatively poorly (Fig. 3). All three transmission models had MCC values > 0.9. Lethality models had lower MCC values relative to transmission models (ranging from 0.7424 to 0.8114), with the combined L1M model more accurate than either standard (L1) or molecular only (LM) models alone. In contrast, morbidity models were not very accurate, with MCC values < 0.5 (0.4416–0.4598) for models trained on any data type.

In conclusion, transmission classification models had the overall highest performance metrics and were very accurate in predictive outcomes when employing internally generated data. Lethality classification models offered similarly high performance with reasonable predictive ability. In contrast, morbidity classification models offered minimal predictive capabilities. Within each classification model, the combined standard and molecular data type models offered the highest predictive value for transmission (T1M) and lethality (L1M), illustrating the usefulness of combining these two types of data for training of ML algorithms.

### Feature importance for each model

**Fig. 4:**
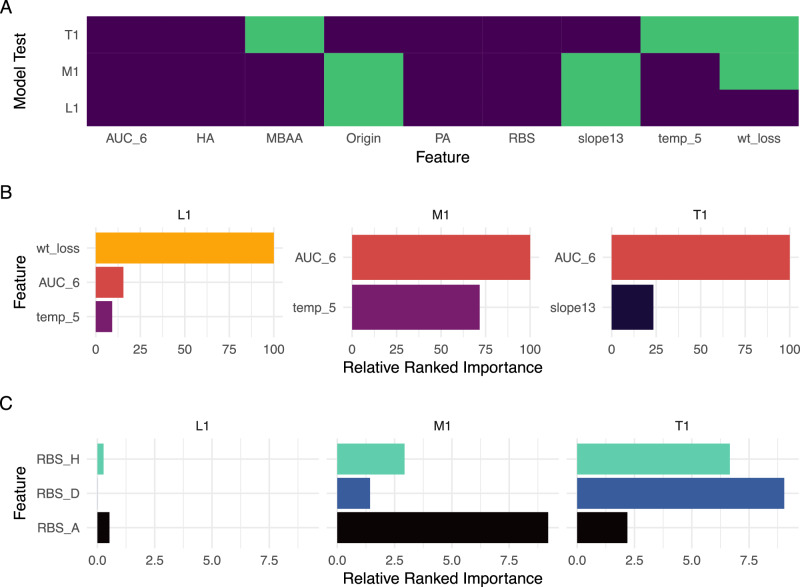
Variability in feature selection among different models employing standard data type. **A** Feature inclusion (purple) or exclusion (green) for lethality (L1), morbidity (M1), and transmission (T1) models employing the standard data type. Individual feature definitions are provided in Supplementary Data 1. **B** Relative ranked importance of top numeric features included in L1, M1, and T1 models. **C** Relative ranked importance of human (H), dual (D), or avian (A) predicted receptor binding preference in L1, M1, and T1 models. Relative ranked performance among features of all models is shown in Supplementary Data 9–14, Supplementary Data 10, 12, and 14 contain ranked importance results for models trained with molecular datasets. Relative ranked importance values are set to 100 for the most important feature and scaled to relative importance for remaining features independently within each model. For each model in (**B**, **C**) features are consistently scaled but separated out for visual purposes.

We next examined in more detail the specific features of each model. As shown in Fig. 4A, all three classification models employing the standard data type (L1, M1, T1) shared several common features (area under the curve days 1–6 [AUC_6], hemagglutinin [HA], polymerase activity [PA], receptor binding preference [RBS], all features defined in Supplementary Data 1), with variability among other features present depending on the classification. Both L1 and M1 models included the absence or presence of a multi-basic amino acid HA cleavage site (MBAA) and a temperature input; in contrast, T1 included features (Origin, slope_1,3_) not present in highest-performing lethality or morbidity models. RBS, PA, HA, and Origin were included in all combined data type models regardless of classification, further highlighting the critical and multifactorial role many of these features contribute to viral pathogenicity and transmissibility outcomes.

Among lethality models, standard and combined models had comparable features, with weight loss followed by MBAA the highest ranked features for both L1 and L1M (Table 2, Fig. 4B) (Supplementary Data 9, 10). In contrast, while included in the highest-performing models, predicted receptor binding preference and HA subtype had minimal contributing impact. Highest importance features of the LM molecular model were HA positions 214 V, 160 T, and 496 R (H3 HA numbering throughout).

Morbidity models also showed similar features of importance across the standard and combined models (Supplementary Data 11, 12). Both M1 and M1M models shared area under the curve of days 1–6 (AUC_6), temperature (temp_5), and MBAA as the three features of highest importance (Table 2). Receptor binding preference, polymerase activity, and HA subtype were less impactful, but notably important. Molecular position HA-227S had the highest importance in the MM molecular only model and was moderately important in the combined model; positions HA-196Q and PB2-627K were also highly ranked features across both molecular and combined models. Like the L1M model, in the combined M1M model, the highest ranked features were derived from in vivo experimentation (AUC_6, temp_5) and not sequential data.

With the transmission standard model (T1), day 1–6 titer area under the curve showed the highest importance, followed by slope_1,3_, H5 subtype, and RBS (Table 2, Fig. 4B); polymerase activity (PA) had minimal influence (Supplementary Data 13–14). For the TM molecular only model, PB2-627E, HA-138A, and HA-21S had the strongest impact. Interestingly, unlike L1M and M1M combined models, the most impactful features of the combined T1M model were derived from molecular-based and not in vivo-derived data (Table 2).

Assessments of relative ranked importance between the three classification models employing similar data types further highlights the variable strength ML algorithms consider different features. AUC_6 was among the top three ranked features across all standard data type classification models but was substantially less critical in the lethality model (L1) than either M1 or T1, where wt_loss was the most important feature (Fig. 4B). Interestingly, among categorical features such as predicted receptor binding preference, models differentially weighted specific variables within a feature (Fig. 4C). For example, while both M1 and T1 models had RBS as a comparable weighted feature across both models (with categorical responses of avian, human, or dual predicted binding), avian binding was highest ranked in the morbidity standard model among the three responses yet lowest ranked in the transmission standard model. Collectively, close attention to which features are included/excluded from different classification models sourced from different data types, as well as the relative ranked importance of features within each model, provides valuable context towards understanding the drivers of the phenotypic outcomes predicted by these ML algorithms.

### Validation of model predictive metrics on simulated and externally generated in vivo data

**Fig. 5:**
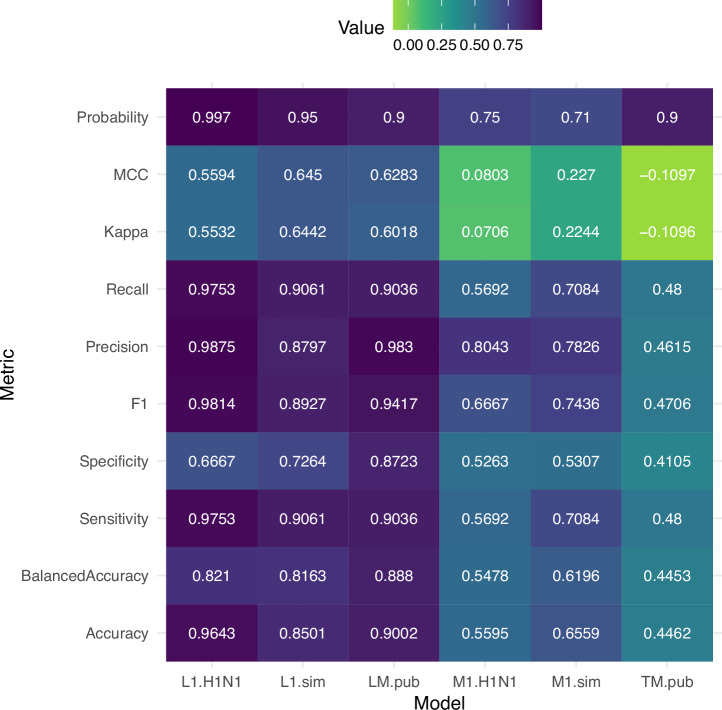
Performance metrics for models tested with externally generated data. Heat map depicting 9 performance metrics and the probability of prediction threshold determined for models including standard data (L1, M1) and molecular only data (LM, TM) from three externally generated datasets (H1N1, sim, pub) as described in Table 2 and the methods. Values range from -1 to 1 (MCC, Kappa) or 0 (worst, green) to 1 (best, purple) (all other metrics).

The findings discussed above support that the lethality and transmission models had high performance metrics trained from our primary dataset of in vivo experimentally-generated data, but it was unknown if this high performance would be maintained when testing data was generated under conditions that diverged from the training data. We first evaluated the performance of models informed by in vivo data metrics (standard lethality and morbidity), by testing data generated from ferret inoculations with two H1N1 IAV from 11 different laboratories (*n* = 88)^24^, or from simulated values based upon our primary dataset (Table 2) (see methods). Overall, performance metrics from the H1N1 (L1-H1N1) and simulated (L1-sim) data were generally comparable to those obtained with our primary dataset, with a noticeable consistency in some and a decrease in other model metrics (Fig. 5). For L1-H1N1, balanced accuracy (0.821) was less than L1 (0.9314), while sensitivity (0.9753) and F1 (0.9814) were higher than the L1 model (0.9235 and 0.9548, respectively). However, specificity (0.6667) had a noticeable drop, also impacting the MCC (0.5594). Metrics for L1-sim were not too dissimilar from L1 but with an increase in specificity (0.7264) and MCC (0.645), and a decrease in sensitivity (0.9061) and F1 (0.8927).

Consistent with other morbidity models, both the H1N1 (M1-H1N1) and simulated (M1-sim) data performed poorly and consistently worse than the M1 model tested with the primary dataset across metrics (Fig. 5). The simulated data, while not very accurate (0.227) performed better over the H1N1 data which was a random prediction (0.0803). These results support that our well performing lethality L1 model (which includes features derived from in vivo experimentation) maintained high performance metrics when data were generated under a consistent protocol in-house or when certain inclusion criteria were met between laboratories providing data, despite limitations in the H1N1 dataset due to limited sample size and viral diversity (Table 2); use of simulated data provided a secondary validation approach to overcome these limitations, despite being inherently unrealistic compared to the primary dataset presented here.

To rigorously evaluate the performance of models informed by molecular features alone (lethality LM, and transmission TM models), we tested these models for lethality (LM-pub) and transmission (TM-pub) with a dataset of previously published data sourced from 68 publications external to our group that employed comparable experimental conditions as our primary dataset (Supplementary Data 18). Strikingly, results from the LM-pub model, tested on ferret lethality outcomes following in vivo experimentation by external research groups only, performed comparably well to all lethality models tested with our primary dataset (Fig. 5). We found comparable high balanced accuracy between LM-pub and LM models (0.888 and 0.9051, respectively) consisting of near equal sensitivity (0.9036 and 0.9617, respectively) and specificity (0.8723 and 0.8485, respectively). The outcome between LM-pub and LM also had a high F1 (0.9417 and 0.967) and a slightly diminished MCC (0.6283 and 0.7424) driven by higher false positives. In contrast, the TM-pub model performed poorly (balanced accuracy, 0.4453) and showed a near random prediction with a slight misclassification bias (MCC, −0.1097) for classification outcomes, suggesting that the TM did not perform well with independent data, likely due to model overfitting on the internal training data. Collectively, we found that our LM, but not TM model, maintained high predictive accuracy with externally generated data from a variety of independent laboratories, underscoring the importance of including external data sets when validating ML models.

## Discussion

Application of ML approaches in public health settings has escalated in recent years, with increasing use of these algorithms to study a diversity of zoonotic pathogens, including respiratory viruses such as IAV and SARS-CoV-2^30^. In this vein, numerous studies have employed ML models to assess considerations regarding pandemic risk of IAV^31–33^. However, these approaches to date have typically employed molecular sequence data and/or epidemiologic data in their assessments. As in vivo models are employed in IAV pandemic risk assessment rubrics^14,15^, we sought to determine the predictive utility of in vivo data generated for this purpose in a ML context. Our study examined three model outcome variables (lethality, morbidity, and transmission), chosen to represent the most key questions addressed by in vivo experimentation using the ferret model in this setting. While using ML approaches to predict phenotypic outcomes itself is not novel, no studies to date have attempted to assess virus transmissibility phenotypic outcomes, nor validated successful ML approaches of lethal outcome with wholly independent data culled from the published literature. We found that ML approaches can offer high predictive value when informed by diverse in vivo-generated data but vary widely in performance metrics and applicability for wider use depending on the classification outcome chosen. Predicated on the exploratory analyses which underlie the choice of parameters selected for inclusion in our ML algorithms^17^, we show the informative role feature selection may contribute towards identifying the most critical values obtained from in vivo risk assessment work. Ultimately, this work provides additional understanding regarding the biological processes governing key phenotypic outcomes of mammalian IAV infection, and supports that inclusion of in vivo-derived parameters may offer refinements and advantages in strengthening the predictive nature of ML approaches in the context of IAV pandemic preparedness.

There are a diversity of supervised classification ML algorithms available for use. We chose 11 models to represent examples of some of the most frequently employed algorithms which employ different strategies for outcome classification^4^. Interestingly, we observed high consistency among our individual top performing and low performing models among the lethality, morbidity, and transmission classification models developed, likely because all models employed similar testing/training source datasets. While an in-depth assessment of subtle differences between the ML algorithms employed lies outside of this study, it is interesting that models based on recursive partitioning & regression trees and a flexible linear regression model were low performing, whereas ensemble learning methods (gradient boosting, random forest) were among the highest performing, with our in vivo-generated datasets. Similarities in our top performing models with the top performers from recent studies assessing phenotypic predictors from IAV genotypic data^6,34^ is of particular note; it will be interesting to see if future studies employing in vivo-derived pathogenicity and transmissibility data are similarly best suited for analysis using these families of algorithms. Data generated from the ferret model has been used in ML settings previously to predict gastrointestinal states (employing k-nearest neighbor and support vector machine)^35^, or to predict phenotypic brain injury (employing an unsupervised method called k-means clustering)^36^; neither study investigated phenotypic outcomes following viral challenge. Of note, Einav and Ma^12^ developed a ML framework of regression random forests that used hemagglutinin titers (generated in part from influenza virus-inoculated ferrets) to predict influenza virus antibody-serum interactions, but did not include additional phenotypic features from in vivo experimentation. They found high predictability between datasets with different methodologies, illustrating the utility of ML to extrapolate unknown data from disparate information, and a framework that could be applied to other features and predictive outcomes.

Most studies employing ML algorithms to assess predictive phenotypic traits following IAV infection have employed molecular data only or been limited to within-host classification outcomes^6,8,9^. A recent study by Jhutty et al. incorporated serially-collected viral load, hematology analyses, and lung cytokine values from IAV-infected mice as inputs to forecast in vivo IAV infection parameters from blood sample data, employing five regression ML algorithms, include a few used herein^13^. However, this study employed one IAV strain only, and conducted independent model validation employing more recent data collection with identical conditions to the testing dataset. Our study is novel in that we tested three phenotypic within-host and between-host outcomes, and generated models trained on primarily molecular, primarily in vivo-generated, or combined data types for each phenotypic outcome. This approach permits head-to-head comparison of feature inclusion and ranked importance across models. Interestingly, while feature selection did vary to some extent, commonalities in highest-performing features were present across many models (Table 2). This is in agreement with previous analyses from our group supporting correlation of many of these metrics (such as AUC_6) with key molecular and/or phenotypic outcomes within an infected ferret host^17^. Additional ML-based studies which identify specific features captured during mammalian risk assessment activities are warranted so that data from these experiments can be most appropriately interpreted in these settings.

Comparison of feature inclusion and relative ranked importance of features retained in finalized models represents a novel approach towards assessing the relative contribution of different discrete data points frequently employed in research-based pandemic risk assessment activities. Viral shedding in NW specimens represents a standard approach to assess within-host viral fitness in the upper respiratory tract of ferrets; our finding that area under the curve days 1–6 (AUC_6) was not only consistently present in high-performing models but also among the highest-ranked feature in these final models when present, supports the capacity of this summary metric (and not other discrete summary metrics of NW viral titer, such as peak titer) to contribute meaningful information to risk assessments of both pathogenicity and transmissibility. Furthermore, the high utility of AUC_6 as a feature across all models supports that IAV capable of sustaining high titers in the mammalian upper respiratory tract for an extended duration of time contributes to both disease severity outcomes (e.g., extended damage of epithelial cells leading to increased inflammatory processes in respiratory tract tissues^37^) and transmission outcomes (e.g., increased shedding of virus into the environment^38^). Similarly, a binary feature specifying HPAI viruses bearing a multibasic amino acid cleavage site (MBAA) was found to be a highly ranked feature in both the standard lethality (L1) and morbidity (M1) models, likely owing to the presence of this molecular signature to facilitate viral spread to extrapulmonary tissues in mammals (see Table 2). Interestingly, while predicted receptor binding preference (RBS) and predicted polymerase activity (PA) were included in the highest-performing L1, M1, and T1 (transmission) standard models, in agreement with the critical role these properties contribute to mammalian adaptation (see Table 1 for inclusion rationale), they were not among the highest ranked features of these models (Table 2), likely supporting the multifactorial role multiple gene segments ultimately contribute to both disease severity and transmission outcomes^39^. Nonetheless, differences in relative ranked importance among how different models weigh different parameters were observed (e.g., the M1 model ranking an avian predicted binding preference above human- or dual-binders, whereas the T1 model ranks human- and dual-binders higher than an avian predicted binding preference, Fig. 4C); compared to viruses with predicted binding to α2-6 linked sialic acids, IAV with predicted binding to α2-3 linked sialic acids are more frequently associated with severe disease outcomes in ferrets^40^, and less frequently associated with transmission outcomes^41^, supporting that classification models for these distinct phenotypic outcomes would differentially weigh these features. However, the predictive (and not explanatory) nature of ML means concurrent exploratory analyses are needed to rigorously assess statistical and/or correlative links between individual features and other biologic metrics pertinent to each classification outcome examined. Correlative features of this data have been explored previously^17^ which informed aspects of this current work. Collectively, the parameters present in final models do appear to be governed by biological sense, and even in underperforming models, offer value in understanding the relative weight and predictive ability of different virological, clinical, and molecular parameters.

We consistently observed that lethality classification models performed better than morbidity models, tested with either internally or externally generated data. It should be noted that lethal events included in this source dataset are all-cause and inclusive of animals which reached humane endpoints for multiple reasons (primarily but not exclusively due to severe weight loss and development of neurological symptoms). As such, it is possible that additional refinement of this binary classification to delineate specific causes of mortality more clearly could further improve the model performance. In contrast, it is challenging to identify enhanced refinement of causes of weight loss during the acute phase of infection due to the complex and multifactorial viral-host interactions that contribute to the magnitude of this clinical sign. However, it could be possible that a different quantifiable metric capturing disease severity beyond peak weight loss (such as rapidity of weight loss, weight loss during a defined period of time post-inoculation, AUC-based and not peak measures of weight loss, etc)^42^ could offer higher predictive value for this parameter. Moreover, the morbidity model could not incorporate lethal outcome as a feature due to concerns about data integrity. This is because defined weight loss thresholds play a significant role in establishing humane endpoint criteria, being correlated with an impact on morbidity^42^. Including lethality as a variable would essentially create a model reciprocal to the ones where weight loss is the primary predictor. Inclusion of additional in vivo-generated parameters (such as lethargy)^43^ in morbidity ML models could yield added benefit.

While lethality classification models exhibited robust performance metrics against wholly independent, previously published testing data, the high-performance metrics of the transmission classification model employing the primary dataset was not maintained in our validation assessments. The poor performance of transmission classification models with independent data nonetheless contributes important information towards continued development of ML approaches in this area, especially when contextualized alongside high performing models. Our work was limited to evaluation of supervised, classification models; additional investigation of the utility of other model approaches to predict transmission outcomes (such as supervised regression models on continuous features or unsupervised clustering methods to group by similarity)^3,4^ could provide added benefit. Furthermore, both morbidity and transmission models employed key features (Fig. 2B) that were captured as categorical (and not continuous) values; there is an ongoing need to investigate the most effective ways to capture in vivo datapoints into discrete responses in the context of ML work^17^. Molecular data incorporated in all models in this study was limited to key positions in the HA and PB2 genes (Table 1); it is possible that these features sufficiently captured key determinants governing lethality, but not transmission, classification outcomes. Meta-analyses of results from ferret respiratory droplet transmission experiments have provided meaningful predictive information^44^, highlighting the potential of this data type to be employed in other data science applications. Despite the failure of the TM-pub model to maintain the high-performance metrics of TM, this work nonetheless supports the need for continued model development in this area.

Several limitations in the source datasets were present. Predicted receptor binding preference and predicted polymerase activity were features included across all highest-performing models (albeit at different ranked importance), which speaks to the role these molecular correlates of virulence and transmissibility contribute to IAV host range and mammalian infection. These categorical variables were employed to summarize underlying sequence data due to the need to consider multiple amino acid residues in these features (which, in the case of receptor binding, differ between virus subtypes included in our analysis). However, these parameters were not independently confirmed in laboratory experiments, which can deviate from predicted sequence identity^45^. Inclusion of a variable encapsulating host origin of each IAV in the study provided an additional feature for which predictive importance could be assessed in our phenotypic classification models. However, considering the diversity of routes the heterogeneous IAV isolates included in the primary dataset crossed species barriers prior to virus isolation and characterization^46^, further research is warranted to investigate the relative role phenotypic outcomes are modulated in mammals following infection with viruses originating from different hosts. Infectious virus in NW specimens was captured with two different titration methods (EID_50_ and PFU), due to IAV strain-specific replicative capacities. While the in vivo experimental inoculation and sampling conditions employed in the source data are reflective of conditions frequently employed in risk assessment settings^24^, the predictive values and key features identified by these models could vary under different protocols (e.g., virus dose, route, and volume of inoculation, and ferret age, sex, and weight)^47–49^. Limitations of ML learning in this study include the need to impute missing values and remove features that had too many gaps, making accurate assessment difficult. While not a limitation in terms of model outcomes, ML models require the same features as inputs for which the model was trained. In our case, molecular diversity was greater in our training set compared to the external validation datasets, which requires the manual addition of dummy features to fill in these gaps. Inclusion of numerous zoonotic IAV with greater genetic diversity in our testing and training datasets, compared to studies limited to human IAV only, represents an additional challenge for establishment of robust ML frameworks^11^. We tested several different ML algorithms and many iterations of features; however, our efforts were not exhaustive, leaving room for model improvements and additional insights.

Our development of three classification models stemming from generally similar training data, yielding three different performance outcomes, illustrates that modeling different phenotypic outcomes informed by in vivo data does not represent a one-size-fits-all approach, and emphasizes the necessity of validating ML performance against independent, real-world data. It should be noted that the models presented here are specific to data obtained in experimentally inoculated ferrets, and results cannot be directly extrapolated to phenotypic outcomes following IAV infection in a different laboratory model species (e.g., mice) or humans. That said, these models nonetheless provide a first step towards greater biological insight and understanding of contributing features to these outcomes that may be retained across scales, and offer immediate benefit towards refining existing ferret studies conducted for the purposes of IAV pandemic risk assessment. Collectively, this study supports that ML algorithms can extract meaningful information from previously conducted work in vivo and offers areas of future refinement to risk assessment studies employing in vivo-generated data, in line with other recent efforts in the field to incorporate novel analytical frameworks into risk assessment activities^50^. Furthermore, this work offers a framework to investigate additional experimental classification outcomes frequently captured in ferret studies (such as viral spread in respiratory tract tissues) and virological metadata (such as NA subtype and full protein sequences) not explicitly assessed here. Inclusion of metagenomic data from virus-infected animals^51^ represents an additional area for which use of ML algorithms have shown utility (when employing human specimens) in predicting clinical outcomes^52^ and could offer added benefit to existing laboratory workflows. As IAV continue to pose a persistent threat to public health, our work highlights that analysis approaches leveraging laboratory data generated for the purposes of risk assessment to improve pandemic risk assessment rubrics are needed and can provide meaningful benefit to the field.

## Methods

### Primary dataset

Male ferrets (*Mustela putorius furo*, Triple F Farms, Sayre, PA) were 5–12 months of age and serologically negative to circulating influenza A and B viruses prior to use. Animals were inoculated with 10^5^ to 10^7^ infectious units of IAV intranasally in a 1 ml volume (minimum *n* = 3 ferrets per virus), and housed inside a HEPA-filtered Duo-Flo BioClean mobile environmental enclosure (Lab Products) for the duration of each experiment. Daily temperature and weight measurements were collected from ferrets post-inoculation (p.i.) as previously described^53^; any animal that lost >25% preinoculation body weight or exhibited signs of neurological involvement was humanely euthanized. Nasal wash (NW) specimens were collected on alternate days p.i. from all ferrets starting day 1 p.i. (85% of all ferrets) or day 2 p.i. and immediately frozen at −80 °C until titration. Viral stock employed for animal inoculations were propagated, and specimen titrations were performed, in either 10–11 day old embryonated hen’s eggs to determine a 50% egg infectious dose (EID_50_) or MDCK cells to determine a plaque forming units titer (PFU) as specified in Supplemental Table 1^54^. All viral titers are reported per milliliter and titration limits of detection were 10^1.5^ EID_50_/ml or 10 PFU/ml. All animal work was approved by CDC’s Institutional Animal Care and Use Committee and conducted in an Association for Assessment and Accreditation of Laboratory Animal Care (AAALAC) International-accredited animal facility. Experiments were conducted at either BSL2 or BSL3 containment, including enhancements, as required by the U.S. Department of Agriculture and the Federal Select Agent Program^55^. We have complied with all relevant ethical regulations for animal use.

Data were aggregated from experiments conducted over approximately 25 years and (with few exceptions) have been published previously (see Supplemental Table 1) and described^17,42^, with source data available at data.cdc.gov^56^. Maximum rise in temperature over preinoculation baseline (temperature range 37–40 °C) between days 1–5 p.i. (temp_5) or days 1-14 p.i. (temp) was reported as a rise in °C. Maximum weight loss below preinoculation baseline (reported as a normalized percentage of weight change), observed over the 14-day experimental period was determined for each animal^42^. Virus transmissibility of wild-type viruses by respiratory droplets was assessed using a strict 1:1 donor:contact ratio on a subset of viruses (96 out of 125, 76.8%) as described previously^57^, with successful transmission defined as both detection of infectious virus and seroconversion to homologous virus in contact animals. Additionally, viruses that exhibited 0% transmissibility in the presence of direct contact were identified as 0% transmissibility by respiratory droplets in this study. All viral titers in this study are presented as log_10_ titer, and all calculations were performed with the log_10_ of the measured virus titer and not the virus titer itself, in agreement with previous work^58^.

Host origin of each virus was identified as avian or mammalian (inclusive of human, swine, and canine) based on virus lineage, not species of isolation. Avian-origin viruses were identified as highly pathogenic avian influenza (HPAI) and possessing a multibasic amino acid (MBAA) HA cleavage site, or not (see Supplemental Table 1). In all ML datasets, HA and PB2 sequences were obtained from the Global Initiative on Sharing All Influenza Data (GISAID) or NCBI Influenza Virus Resource and HAs were aligned with A/Aichi/2/1968 (H3N2) HA sequence; viral stocks employed for ferret inoculation were routinely sequenced to confirm identity with consensus as is specified in study-specific references in Supplemental Table 1. 33 HA and 5 PB2 amino acids were selected due to known roles in host adaptation, receptor binding preference, and polymerase activity^39,59^. Predicted receptor binding profile was defined based on hemagglutinin (HA) amino acid residues as avian (190E, 225 G [H1 only], 226Q, 228 G [all other subtypes]), human (190D/N, 225D [H1 only], 226 L/V/I, 228 S [H2, H3 only]), or dual (190D/E/A, 225 G/D/N/E [H1 only], 226 L, 228 G [all other subtypes]), with few exceptions (see Supplemental Table 1). Predicted polymerase activity was defined based on polymerase basic 2 (PB2) amino acid residues as human origin (the presence of at least one of 590 S/591 R, 627 K, or 701 N) or avian origin (not meeting these criteria).

Ferrets (*n* = 717) were inoculated with an expansive panel of 125 influenza A viruses by standard high-dose intranasal administration, as previously described^17^ and specified in Supplemental Table 1. Animals were inoculated with H1 (*n* = 213 ferrets, 29.71%), H2 (51, 7.11%), H3 (123, 17.15%), H5 (156, 21.76%), H7 (149, 20.78%), and H9 (25, 3.48%) hemagglutinin subtype viruses. Strains in this study were inclusive of both avian (*n* = 378 ferrets, 52.72%) and mammalian (n = 339, 47.28%) origins and encapsulated viruses derived from wild bird surveillance, gallinaceous poultry outbreaks, and confirmed human infections; avian-origin viruses were classified as highly pathogenic avian influenza (HPAI) (*n* = 207 ferrets, 54.76%) or low pathogenicity (171, 45.24%). All ferrets in the study were productively infected with virus, as determined by the presence of infectious virus in serially collected nasal wash (NW) specimens. Animals were observed daily for 14 days post-inoculation unless they met criteria for humane euthanasia and did not survive the observation period (102 ferrets, 14.22%). Overview of the ML analysis workflow employed is presented in Fig. 1.

### Statistics and reproducibility

Data were collected from a minimum of three ferrets per virus, per treatment group as detailed in the Primary Dataset section above. Sample sizes and replicates are detailed in Table 2 and Supplementary Data files. Specific analyses are presented in relevant methods sections below.

### Data preprocessing and partitioning

All analyses were conducting using R v4.2.1^60^ using the additional packages tidyverse v1.3.2^61^, ggplot2 v3.4.0^62^, funModeling v1.9.4^63^, and caretEnsemble v2.0.1^64^ beyond those cited below. Data were imported into R in CSV format and cleaned and processed to make each column a feature, and each row an observation. A binary column for respiratory transmission was created as yes when respiratory titer and serology were both yes, else it was no. This was then converted to numeric (1, 0) and a proportion of transmission on a per virus basis was calculated. Viruses with ≥50% transmission among ferrets were scored as yes, else no. Weight loss categories were chosen from weight loss values as less than 5% change being ‘none’, then the remaining being equally divided into thirds for low (5–9.5%), medium (9.5–14.5%), and high (14.5–27.6%); weight loss ≥15% is frequently detected in ferrets with elevated disease severity, with these animals more likely to continue to lose weight and reach humane endpoints relative to ferrets in low or medium weight loss categories which more frequently regain weight by the end of the observation period^42^. The growth or decay rates of ferret NW titer between days 1 and 3 p.i. (termed slope_1,3_) was computed by subtracting the log_10_ of day 3 titer from the log_10_ of day 1 titer on a per ferret basis, and dividing by 48 h, as previously described^58^.

Missing data values were filled in for weight loss and temperature with the median value on a per virus per unit basis. Nasal wash titer values for non-standard collection days (2,4,6,8 p.i.) were imputed as the mean of the standard collection days (1,3,5,7,9 p.i.) immediately preceding and following. Missing values for lung titers that were tested were imputed with the respective limit of detection (LOD) for titration matrix (1.5 for egg, 1 for cell). Peak NW titers for each ferret (detected between days 1–5 p.i.) were determined, and area under the curve (AUC) from serially collected NW specimens (between days 1–9 p.i.) per virus and per titration units. As some viruses were associated with lethal outcomes in ferrets, NW specimens from at least two ferrets per sampling timepoint were required to conduct the AUC, otherwise the virus was excluded from analysis. We used the AUC function in DescTools v0.99.47^65^ to calculate using the trapezoid method with 1000 bootstraps to obtain percentile confidence intervals at 95%.

Missing molecular amino acid signatures were filled in with the dummy variable ‘Z’. Non-numeric predictor variables were one-hot encoded using the R package fastDummies v1.6.3^66^ and the data set was split using rsample v1.1.1^67^ into training (70%) and testing (30%) data sets. Training and testing data were inspected to ensure outcome variable balance/proportion was consistent with the full dataset. The R package caret v6.0.93^68^ was used to perform ML using the trainControl function with repeated cross validation of 10 folds and 2 repeats (total of 20x) saving results as a twoClassSummary on the training/testing split data. Additionally, the training data utilized the preProcess option in the train function to scale and center the data, while removing near zero variables for ML algorithms to function properly.

### Inputs and outputs—feature selection

We examined three overarching classification models to classify and predict a binary outcome (Table 1). Lethality, to predict mortality as a yes or no, defined as a ferret not surviving the 14-day experimental period for any reason. Morbidity, determined as a measure of high proportion body weight lost (≥14.5%) or not. Transmission, to predict a ferret likely to transmit in a respiratory droplet transmission model (≥ or <50% transmission as yes or no), defined as a contact animal seroconverting to homologous virus with infectious virus detected in at least one collected NW specimen post-contact. Predictive outcomes are made on a per ferret basis for all models, however, for transmission outcome designation was based on a per virus basis, i.e., (≥ or <50% transmission).

For each model, three sub-models were tested employing different sets of input data (Tables 1 and 2, Supplementary Data 1): either molecular data alone consisting of 33 HA and 4 PB2 amino acid features, in vivo-derived data with limited key molecular data (standard) consisting of 7 numeric clinical features and 7 categorical viral features, and a combined model employing all molecular and in vivo-derived parameters. As a starting point we used backwards feature selection with the caret function rfe in R. However, we largely relied upon biological subject matter expertise (supported by the published literature) in selection of initial model features, and model performance evaluation to refine (add or subtract) certain features (Supplementary Data 1). For area under the curve (AUC), in addition to initial ML feature testing, we evaluated the various AUC inclusive days with predictive power scores using the R package ppsr v0.0.2^69^ showing AUC of days 1–6 to be the most predictive for lethality and morbidity models (Supplementary Data 15) which become the constant AUC feature used.

### Models tested and evaluation

For each model we tested eleven ML algorithms: generalized linear model (glm), logistic regression GLM (glmnet), Bayesian GLM (bayesglm), boosted logistic regression (BlogReg), k-nearest neighbor (knn), neural network (nnet), stochastic gradient boosting (gbm), recursive partitioning and regression decision tree (rpart), support vector machine with radial basis function kernel (svm), and two random forest methods (rf, ranger). In certain instances where model performance was suboptimal, we used the caretStack function with the rf method to generate stacked or ensemble models from the top few performing models.

Model results were evaluated by examining a series of metrics with balanced accuracy being the primary focus, but all metrics were examined for consistency in high performance when choosing a top model as a quality control check. We also looked at the underlining metrics to balanced accuracy by examining sensitivity and specificity. In cases where two models had similar balanced accuracy, we chose models that were more equal in sensitivity and specificity than one exhibiting more skew. Sensitivity is the true positive rate, or percentage of positive cases the model can detect correctly. Specificity is the true negative rate, or the percentage of negative cases the model can detect accurately. Balanced accuracy is the average of sensitivity and specificity. When balanced accuracy was similar between two models, sensitivity and specificity would be examined more closely to identify reasonable balance, by selecting models with a little less sensitivity if the specificity could be increased. This is because in our models the ‘positive’ case was the more abundant variable (i.e., no) and ‘negative’ case was the minority, which was the case of most interest (see Obs. In Table 2 for class imbalance). However, initial examination and testing of the data with resampling methods (SMOTE^70^ and ROSE^71^) did not enhance performance with artificial rebalancing and therefore did not warrant inclusion. Furthermore, as overall models perform well without, balancing was not necessary with these data.

When a final algorithm and set of features for a model had been determined via model selection it was further optimized via hyperparameter tuning using balanced accuracy to establish the final model. In addition to the standard metrics, we also examined Matthew’s Correlation Coefficient (MCC) as a post-assessment for final model evaluation, which is a balanced and symmetric measure of all four confusion matrix categories (i.e., true and false, positives and negatives) which examines the correlation between observed and predicted values. MCC range is from −1 (complete misclassification) to 1 (perfect classification). Zero values mean random chance.

We used the varImp function from the caret package to compile relative ranked importance of variables. Tree based models calculate variable importance based on how often a particular predictor is selected for splitting and how much it improves the purity or homogeneity of the resulting subsets. None tree based measure (e.g., nnet) utilize other methods such as permutation importance or partial dependence plots. Importance scores were scaled from 0 to 100 with the most frequently important variable set at 100. This method provides a relative rank of importance within a model and numeric values are not meant to be compared across different models.

### Model validation with externally generated data

A simulated dataset was generated from parameters in the primary dataset after missing data imputation. Focusing on the features that were important in the various models tested, we grouped the primary data set by hemagglutinin subtype (HA), high pathogenic avian influenza multi-basic amino acid (MBAA), lethality, and predicted receptor binding/polymerase activity (RBS/PA). We then calculated the mean and standard deviation using the summarize function in dplyr v1.0.10^72^. A few group values were manually converted from zero or NA to fill in gaps (Supplementary Data 16). We used this information along with categorical features to set up variable definitions, then used the simstudy package v0.6.0^73^ to simulate the data by group. All group combinations were combined into a single data frame, representative of the primary dataset features of interest. This was performed to create a lethality-severity simulated dataset with 2900 observations, equally balanced with 100 observations per group (Supplementary Data 17).

All raw data informing the H1N1 dataset (employing a low pathogenic avian influenza virus and a 2009 pandemic-derived virus) was published previously^24^. Ferrets were inoculated with 500 µl of each virus at a challenge dose of 10^6^ PFU. Results from 11 independent groups are captured (CDC was one of the 11 groups, but data from this study is not included in the internally generated datasets shown in Supplementary Table 1). Studies included in the aggregated dataset employed for external validation of lethality (LM) and transmission (TM) molecular models met the following criteria: source publications were PubMed-indexed, ferrets were healthy and serologically naïve to circulating IAV prior to inoculation, inoculation was performed with a high (≥10^5^–10^7^ infectious units) dose of wild-type IAV delivered intranasally in a 0.5–1 ml volume, and sequence data matching the virus strain name was publicly available (NCBI or GISAID) (Supplementary Data 18). Lethality and transmission outcome were aggregated on a per-ferret basis. Lethality was all-cause based on the specific humane endpoint criteria of the research group conducting the work, which may vary between institutions. For transmission studies, transmission was defined as both detection of infectious virus in contact animals and seroconversion to homologous virus (which may differ to how source publications reported transmission events), in a respiratory droplet setting with or without directional airflow, and only included transmission outcomes when a strict 1:1 donor:contact ratio was employed.

### Reporting summary

Further information on research design is available in the Nature Portfolio Reporting Summary linked to this article.

### Supplementary information

Supplementary Information

Description of Additional Supplementary Files

Supplementary Data 1-18

Reporting Summary

## Data Availability

Data is available from data.cdc.gov under the title “An aggregated dataset of serially collected influenza A virus morbidity and titer measurements from virus-infected ferrets”. A companion data descriptor providing more data is published^56^. Supplementary Data files 1-18 are presented. Supplementary Data 1: Table of features tested in each iteration of model testing. Includes the classification model (lethality, morbidity, transmission), the type of dataset used for testing (standard, combined, molecular), the outcome being classified. Descriptors of each feature are provided below. Supplementary Data 2: Table of all 14 evaluation metrics, for all 11 machine learning algorithms tested, for each iterative test of lethality classification models on the internal standard dataset. Supplementary Data 3: Table of all 14 evaluation metrics, for all 11 machine learning algorithms tested, for each iterative test of lethality classification models on the internal molecular only and combined datasets. Supplementary Data 4: Table of all 14 evaluation metrics, for all 11 machine learning algorithms tested, for each iterative test of morbidity classification models on the internal standard dataset. Supplementary Data 5: Table of all 14 evaluation metrics, for all 11 machine learning algorithms tested, for each iterative test of morbidity classification models on the internal molecular only and combined datasets. Supplementary Data 6: Table of all 14 evaluation metrics, for all 11 machine learning algorithms tested, for each iterative test of transmission classification models on the internal standard dataset. Supplementary Data 7: Table of all 14 evaluation metrics, for all 11 machine learning algorithms tested, for each iterative test of transmission classification models on the internal molecular only and combined datasets. Supplementary Data 8: Table of final model parameters used for each top model showing the machine learning algorithm, and the relevant parameters that were tuned with the final value for each. Supplementary Data 9: Table of feature importance for each iterative test of lethality classification models on the internal standard dataset. The top importance value is scaled to 100 and remaining features are relatively ranked accordingly. Supplementary Data 10: Table of feature importance for each iterative test of lethality classification models on the internal molecular only and combined datasets. The top importance value is scaled to 100 and remaining features are relatively ranked accordingly. Supplementary Data 11: Table of feature importance for each iterative test of morbidity classification models on the internal standard dataset. The top importance value is scaled to 100 and remaining features are relatively ranked accordingly. Supplementary Data 12: Table of feature importance for each iterative test of morbidity classification models on the internal molecular only and combined datasets. The top importance value is scaled to 100 and remaining features are relatively ranked accordingly. Supplementary Data 13: Table of feature importance for each iterative test of transmission classification models on the internal standard dataset. The top importance value is scaled to 100 and remaining features are relatively ranked accordingly. Supplementary Data 14: Table of feature importance for each iterative test of transmission classification models on the internal molecular only and combined datasets. The top importance value is scaled to 100 and remaining features are relatively ranked accordingly. Supplementary Data 15: Table of predictive power scores for selected features against the outcome variable (y). Supplementary Data 16: Table of summary statistics from the internal dataset grouped by HA, MBAA, RBA-PBS combination, and lethality outcome used to create simulated dataset. The mean (m), standard deviation (sd), skew (sk), and kurtosis (kr) are shown for the features wt_loss, tem5, and AUC_6. Supplementary Data 17: Table of the 2900 simulated data observations. Supplementary Data 18: Table of influenza A viruses from published literature as an external validation dataset to test the molecular only based models for lethality and transmission. Includes virus metadata, and source references with number of observations used for lethality and transmission classification models.

## Code availability

R code used for machine learning analyses and figures is available on Github (https://github.com/CDCgov/machine-learning-influenza-ferret-model).
