# Supplementary material for: Machine learning approaches for influenza A virus risk assessment identifies predictive correlates using ferret model in vivo data
Source: Commun Biol. 2024 Aug 1;7:927. doi: 10.1038/s42003-024-06629-0 (PMC11294530; doi:10.1038/s42003-024-06629-0)
Supplement: Supplementary file 1 — Supplementary Information [file 42003_2024_6629_MOESM1_ESM.pdf]

Supplemental Table 1. Influenza A viruses evaluated in the ferret model.

Virus Name	Subtype[a]	Titration units[b]	RB[c]	PA[d]	Full seq[e]	References[f]
A/duck/Alberta/35/1976	H1N1	EID_{50}	A	A	yes	(1)
A/turkey/Kansas/4880/1980	H1N1	EID_{50}	D	A	yes	(1)
A/turkey/South Dakota/7034/1986	H1N1	EID_{50}	A	A	yes	(1)
A/duck/New York/15024-21/1996	H1N1	EID_{50}	A	A	no	(1)
A/mallard/Alberta/119/1998	H1N1	EID_{50}	A	A	yes	(1)
A/New Caledonia/20/1999	H1N1	EID_{50}	H	H	yes	(2)
A/Solomon Islands/03/2006	H1N1	EID_{50}, PFU	H	H	yes	(1), here
A/Brisbane/59/2007	H1N1	PFU	H	H	yes	(3)
A/South Dakota/6/2007	H1N1	EID_{50}	H	H	yes	(4)
A/California/4/2009	H1N1pdm09	PFU	H	H	yes	(3, 5, 6)
A/California/7/2009	H1N1pdm09	PFU	H	H	yes	(7)
A/Mexico/4482/2009	H1N1pdm09	EID_{50}, PFU	H	H	yes	(3, 4, 8, 9)
A/New York/1682/2009	H1N1pdm09	PFU	H	H	yes	(10)
A/Texas/15/2009	H1N1pdm09	PFU	H	H	yes	(3)
A/Netherlands/1132/2009	H1N1pdm09	PFU	H	H	yes	(4)
A/Michigan/45/2015	H1N1pdm09	PFU	H	H	yes	(11)
A/Idaho/7/2018	H1N1pdm09	PFU	H	H	yes	(11)
A/Nebraska/14/2019	H1N1pdm09	PFU	D	H	yes	(11)
A/Ohio/2/2007	H1N1v	PFU	H	H	yes	(12)
A/Texas/14/2008	H1N1v	PFU	D	H	yes	(12)
A/Iowa/39/2015	H1N1v	PFU	H	H	yes	(7)
A/Ohio/9/2015	H1N1v	PFU	D	H	yes	(7)
A/Hunan/42443/2015	H1N1v	PFU	D	A	yes	(13)
A/Michigan/288/2019	H1N1v	PFU	H	H	yes	(11)
A/Minnesota/19/2011	H1N2v	PFU	H	H	yes	(14)
A/Minnesota/45/2016	H1N2v	PFU	H	H	yes	(14)
A/Wisconsin/71/2016	H1N2v	PFU	H	H	yes	(14)
A/Ohio/24/2017	H1N2v	PFU	D	H	yes	(11)
A/California/62/2018	H1N2v	PFU	D	H	yes	(11)
A/Alberta/01/2020	H1N2v	PFU	H	H	yes	(11)
A/El Salvador/2/1957	H2N2	EID_{50}	A	H	yes	(15)
A/Albany/6/1958	H2N2	EID_{50}	H	H	yes	(15)
A/England/10/1967	H2N2	EID_{50}	H	H	yes	(16)
A/mallard/New York/6750/1978	H2N2	EID_{50}	A	A	yes	(15)
A/mallard/Maryland/235/2001	H2N3	EID_{50}	A	A	yes	(16)
A/chicken/Pennsylvania/298101-4/2004	H2N2	EID_{50}	A	A	yes	(16)
A/swine/Missouri/2124514/2006	H2N3	EID_{50}	D	H	yes	(16)
A/swine/Missouri/4296424/2006	H2N3	EID_{50}	D	H	yes	(2)
A/Victoria/3/1975	H3N2	EID_{50}	H	H	yes	(17)
A/Nanchang/933/1995	H3N2	EID_{50}	H	H	yes	(18)
A/Panama/2007/1999	H3N2	EID_{50}, PFU	H	H	yes	(17), here
A/Wyoming/3/2003	H3N2	PFU	H	H	yes	(19)
A/New York/55/2004	H3N2	EID_{50}	H	H	yes	(2)
A/Wisconsin/67/2005	H3N2	PFU	H	H	yes	here
A/Perth/16/2009	H3N2	PFU	H	H	yes	(20, 21)
A/Texas/50/2012	H3N2	PFU	H	H	yes	(22)

A/canine/Illinois/12191/2015	H3N2	EID_{50}	A	A	yes	(23)
A/swine/Oklahoma/A02218157/2017	H3N2	PFU	H	H	yes	(24)
A/Kansas/13/2009	H3N2v	PFU	H	H	yes	(25)
A/Minnesota/11/2010	H3N2v	EID_{50}, PFU	H	H	yes	(25)
A/Pennsylvania/14/2010	H3N2v	PFU	H	H	yes	(25)
A/Indiana/8/2011	H3N2v	PFU	H	H	yes	(20, 21, 25)
A/Iowa/8/2011	H3N2v	PFU	H	H	yes	(26)
A/Ohio/13/2012	H3N2v	PFU	H	H	yes	(26)
A/Michigan/39/2015	H3N2v	PFU	H	H	yes	(26)
A/Ohio/27/2016	H3N2v	PFU	H	H	yes	(26)
A/Ohio/13/2017	H3N2v	PFU	H	H	yes	(24)
A/Hawaii/28/2020	H3N2v	PFU	H	H	yes	(24)
A/Hong Kong/486/1997	HPAI H5N1	EID_{50}	A	A	yes	(17, 27)
A/goose/Vietnam/113/2001	HPAI H5N1	EID_{50}	A	A	no	(28)
A/chicken/Indonesia/7/2003	HPAI H5N1	EID_{50}	A	A	yes	(27)
A/chicken/Korea/ES/2003	HPAI H5N1	EID_{50}	A	A	yes	(27)
A/Hong Kong/213/2003	HPAI H5N1	EID_{50}	A	A	yes	(17)
A/chicken/Vietnam/NCVD/31/2004	HPAI H5N1	EID_{50}	A	A	no	(27)
A/Thailand/16/2004	HPAI H5N1	EID_{50}	A	H	yes	(19, 27)
A/Thailand/Kan/353/2004	HPAI H5N1	EID_{50}	A	A	yes	(27)
A/Thailand/SP83/2004	HPAI H5N1	EID_{50}	A	A	yes	(27)
A/Vietnam/1203/2004	HPAI H5N1	EID_{50}	A	H	yes	(2, 27, 29-31)
A/Vietnam/1204/2004	HPAI H5N1	EID_{50}	A	A	yes	(27)
A/Indonesia/5/2005	HPAI H5N1	EID_{50}	A	A	yes	(17, 27, 32)
A/Vietnam/HN30408/2005	HPAI H5N1	EID_{50}	A	A	no	(17)
A/chicken/Korea/IS/2006	HPAI H5N1	EID_{50}	A	A	no	(33)
A/Indonesia/CDC625/2006	HPAI H5N1	EID_{50}	A	A	yes	here
A/Egypt/2321-NAMRU3/2007	HPAI H5N1	EID_{50}	A	A	yes	(33)
A/chicken/Korea/Gimje/2008	HPAI H5N1	EID_{50}	A	A	no	here
A/Egypt/4935-NAMRU3/2009	HPAI H5N1	EID_{50}	A	H	yes	(33)
A/BarnSwallow/Hong Kong/D10-1161/2010	HPAI H5N1	EID_{50}	A	A	yes	(33)
A/Egypt/N03072/2010	HPAI H5N1	EID_{50}	A	H	yes	(33)
A/Bangladesh/5487/2011	HPAI H5N1	EID_{50}	A	H	yes	(33)
A/duck/Vietnam/NCVD-672/2011	HPAI H5N1	EID_{50}	A	A	yes	(33)
A/duck/Vietnam/NCVD-1206/2012	HPAI H5N1	EID_{50}	A	A	yes	(33)
A/duck/Vietnam/NCVD-1232/2012	HPAI H5N1	EID_{50}	A	A	yes	(33)
A/Vietnam/VP12-3/2012	HPAI H5N1	EID_{50}	A	H	yes	(34)
A/Cambodia/X0123311/2013	HPAI H5N1	EID_{50}	A	A	yes	here
A/Cambodia/X0810301/2013	HPAI H5N1	EID_{50}	A	A	yes	here
A/duck/Vietnam/NCVD-0004/2013	HPAI H5N1	EID_{50}	A	A	yes	(33)
A/duck/Vietnam/NCVD-2848/2013	HPAI H5N1	EID_{50}	A	A	yes	(33)
A/Vietnam/VP13-28H/2013	HPAI H5N1	EID_{50}	A	A	yes	(34)
A/Alberta/1/2014	HPAI H5N1	EID_{50}	A	A	yes	here
A/Am Wigeon/SC/22-000345-001/2021	HPAI H5N1	EID_{50}	A	A	yes	(35)
A/Northern Pintail/Washington/40964/2014	HPAI H5N2	EID_{50}	A	A	yes	(36)
A/turkey/Minnesota/10915/2015	HPAI H5N2	EID_{50}	A	A	yes	(37)
A/Sichuan/26221/2014	HPAI H5N6	EID_{50}	A	H	yes	(37)
A/Yunnan/14563/2015	HPAI H5N6	EID_{50}	A	H	yes	(37)
A/duck/Bangladesh/19D770/2017	HPAI H5N6	EID_{50}	A	A	yes	(37)

A/gyrfalcon/Washington/41088-6/2014	HPAI H5N8	EID_{50}	A	A	yes	(36)
A/chicken/Texas/18-007912-2/2018	LPAI H7N1	EID_{50}	A	A	yes	(38)
A/turkey/Virginia/4529/2002	LPAI H7N2	EID_{50}	D	A	yes	(39, 40)
A/chicken/Connecticut/260413-2/2003	LPAI H7N2	EID_{50}	D	A	yes	(40)
A/New York/107/2003	LPAI H7N2	EID_{50}	D	A	yes	(2, 30, 39, 40)
A/New York/108/2016	LPAI H7N2	EID_{50}	D	A	yes	(41)
A/Canada/504/2004	HPAI* H7N3	EID_{50}	A	A	yes	(40)
A/Mexico/7218/2012	HPAI* H7N3	EID_{50}	A	A	yes	(42)
A/turkey/California/18-031151-4/2018	LPAI H7N3	EID_{50}	A	A	yes	(38)
A/Netherlands/219/2003	HPAI H7N7	EID_{50}	A	H	yes	(39, 40)
A/Netherlands/230/2003	HPAI H7N7	EID_{50}	A	A	yes	(39, 40)
A/Italy/3/2013	HPAI H7N7	EID_{50}	A	A	yes	(43)
A/turkey/Indiana/1403/2016	HPAI H7N8	EID_{50}	A	A	yes	(44)
A/turkey/Indiana/1573-2/2016	LPAI H7N8	EID_{50}	A	A	yes	(44)
A/shoveler/Egypt/00215-NAMRU3/2007	LPAI H7N9	PFU	A	A	yes	(45)
A/goose/Nebraska/17096-1/2011	LPAI H7N9	EID_{50}	A	H	yes	(42)
A/Anhui/1/2013	LPAI H7N9 (1)	PFU	D	H	yes	(22)
A/Shanghai/1/2013	LPAI H7N9 (1)	PFU	A	H	yes	(22)
A/Taiwan/1/2013	LPAI H7N9 (1)	PFU	A	H	yes	(45)
A/Hong Kong/5942/2013	LPAI H7N9 (2)	PFU	D	H	yes	(45)
A/British Columbia/1/2015	LPAI H7N9 (3)	PFU	D	H	yes	(45)
A/Hong Kong/4553/2016	LPAI H7N9 (5)	EID_{50}	D	H	yes	(46)
A/Guangdong/17SF003/2016	HPAI H7N9 (5)	EID_{50}	A	A	yes	(46)
A/Taiwan/1/2017	HPAI H7N9 (5)	EID_{50}	A	H	yes	(46)
A/chicken/Tennessee/17-007147-2/2017	HPAI* H7N9	EID_{50}	A	A	yes	(47)
A/chicken/Tennessee/17-007431-3/2017	LPAI H7N9	EID_{50}	A	A	yes	(47)
A/Hong Kong/1073/1999	LPAI H9N2	PFU	D	A	yes	(48)
A/Hong Kong/33982/2009	LPAI H9N2	EID_{50}	A	H	yes	(30)
A/Hong Kong/308/2014	LPAI H9N2	EID_{50}	D	A	yes	(48)
A/Anhui-Lujiang/39/2018	LPAI H9N2	EID_{50}	D	A	yes	(48)

[a] H1N1pdm09, denotes viruses isolated from humans after the 2009 H1N1 pandemic. v, denotes variant virus (human infection with swine-origin virus). Epidemiological wave from which H7N9 viruses were isolated from humans is indicated in parentheses. HPAI, highly pathogenic avian influenza virus; LPAI, low pathogenic avian influenza virus. Asterisk denotes HPAI viruses that do not possess a classic multibasic amino acid (MBAA) cleavage site. [b]The method of titration for detection of infectious virus. EID_{50}, 50% egg infectious dose; PFU, plaque forming units in Madin Darby Canine Kidney (MDCK) cells. [c]RB, predicted receptor binding preference. All viruses in this dataset were classified as having a predicted α2-3 (avian-like, A), α2-6 (human-like, H), or mixed (dual, D) sialic acid binding preference defined based on HA amino acid residues as avian (190E, 225G [H1 only], 226Q, 228G [all other subtypes]), human (190D/N, 225D [H1 only], 226L/V/I, 228S [H2, H3 only]), or dual (190D/E/A, 225G/D/N/E [H1 only], 226L, 228G [all other subtypes]); H7 subtype viruses with a deletion from 221-228 aa (H3 numbering) were classified as dual (49). [d]PA, predicted polymerase activity. Viruses were classified as having avian-like (A) or human-like (H) polymerase activity based on PB2 amino acid residues (H, the presence of at least one of 590S/591R, 627K, or 701N; A, not meeting these criteria). [e]Column specifies if full HA sequence was included in molecular-only and combined models. [f]Where applicable, references to previously published data for each virus.

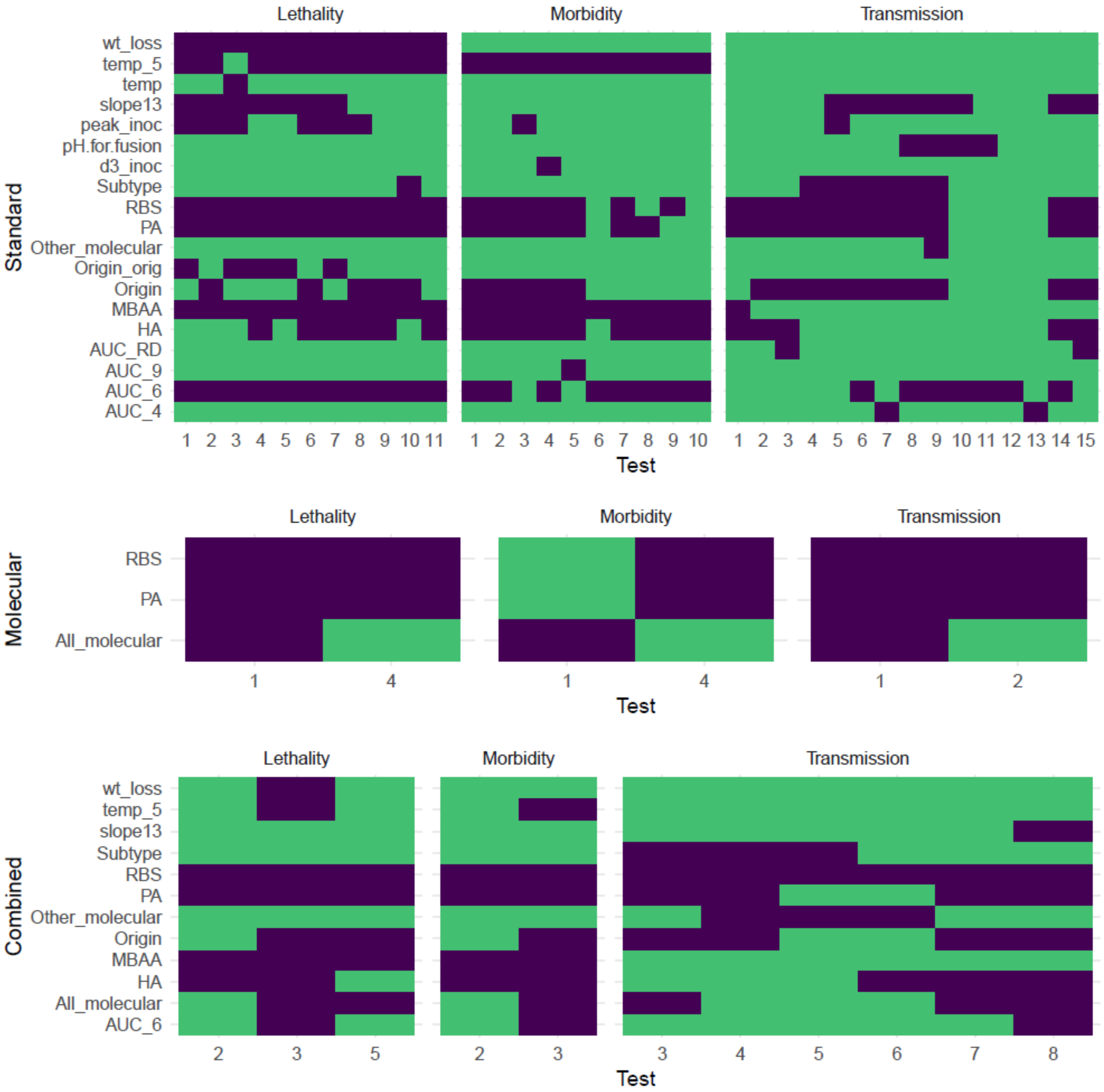

Supplemental Figure 1. Feature inclusion for all supervised classification models trained and tested in this study. Purple, feature inclusion; green, feature exclusion. Feature table reflects data presented in Supplemental Table 2. Feature definitions reported in Table 1 and Supplemental Table 2.

References for Supplemental Table 1.

1. Van Hoeven N, Pappas C, Belser JA, Maines TR, Zeng H, Garcia-Sastre A, Sasisekharan R, Katz JM, Tumpey TM. 2009. Human HA and polymerase subunit PB2 proteins confer transmission of an avian influenza virus through the air. Proc Natl Acad Sci U S A 106:3366-71.
2. Pushko P, Pearce MB, Ahmad A, Tretyakova I, Smith G, Belser JA, Tumpey TM. 2011. Influenza virus-like particle can accommodate multiple subtypes of hemagglutinin and protect from multiple influenza types and subtypes. Vaccine 29:5911-8.
3. Maines TR, Jayaraman A, Belser JA, Wadford DA, Pappas C, Zeng H, Gustin KM, Pearce MB, Viswanathan K, Shriver ZH, Raman R, Cox NJ, Sasisekharan R, Katz JM, Tumpey TM. 2009. Transmission and pathogenesis of swine-origin 2009 A(H1N1) influenza viruses in ferrets and mice. Science 325:484-7.
4. Pearce MB, Belser JA, Houser KV, Katz JM, Tumpey TM. 2011. Efficacy of seasonal live attenuated influenza vaccine against virus replication and transmission of a pandemic 2009 H1N1 virus in ferrets. Vaccine 29:2887-94.
5. Belser JA, Jayaraman A, Raman R, Pappas C, Zeng H, Cox NJ, Katz JM, Sasisekharan R, Tumpey TM. 2011. Effect of D222G mutation in the hemagglutinin protein on receptor binding, pathogenesis and transmissibility of the 2009 pandemic H1N1 influenza virus. PLoS One 6:e25091.
6. Jayaraman A, Pappas C, Raman R, Belser JA, Viswanathan K, Shriver Z, Tumpey TM, Sasisekharan R. 2011. A single base-pair change in 2009 H1N1 hemagglutinin increases human receptor affinity and leads to efficient airborne viral transmission in ferrets. PLoS One 6:e17616.
7. Pulit-Penaloza JA, Jones J, Sun X, Jang Y, Thor S, Belser JA, Zanders N, Creager HM, Ridenour C, Wang L, Stark TJ, Garten R, Chen LM, Barnes J, Tumpey TM, Wentworth DE, Maines TR, Davis CT. 2018. Antigenically Diverse Swine Origin H1N1 Variant Influenza Viruses Exhibit Differential Ferret Pathogenesis and Transmission Phenotypes. J Virol 92.
8. Pushko P, Kort T, Nathan M, Pearce MB, Smith G, Tumpey TM. 2010. Recombinant H1N1 virus-like particle vaccine elicits protective immunity in ferrets against the 2009 pandemic H1N1 influenza virus. Vaccine 28:4771-6.
9. Zhou B, Li Y, Belser JA, Pearce MB, Schmolke M, Subba AX, Shi Z, Zaki SR, Blau DM, Garcia-Sastre A, Tumpey TM, Wentworth DE. 2010. NS-based live attenuated H1N1 pandemic vaccines protect mice and ferrets. Vaccine 28:8015-25.
10. Zhou B, Pearce MB, Li Y, Wang J, Mason RJ, Tumpey TM, Wentworth DE. 2013. Asparagine substitution at PB2 residue 701 enhances the replication, pathogenicity, and transmission of the 2009 pandemic H1N1 influenza A virus. PLoS One 8:e67616.
11. Pulit-Penaloza JA, Brock N, Jones J, Belser JA, Jang Y, Sun X, Thor S, Pappas C, Zanders N, Tumpey TM, Davis CT, Maines TR. 2022. Pathogenesis and transmission of human seasonal and swine-origin A(H1) influenza viruses in the ferret model. Emerg Microbes Infect 11:1452-1459.
12. Belser JA, Gustin KM, Maines TR, Blau DM, Zaki SR, Katz JM, Tumpey TM. 2011. Pathogenesis and transmission of triple-reassortant swine H1N1 influenza viruses isolated before the 2009 H1N1 pandemic. J Virol 85:1563-72.
13. Pulit-Penaloza JA, Belser JA, Tumpey TM, Maines TR. 2019. Mammalian pathogenicity and transmissibility of a reassortant Eurasian avian-like A(H1N1v) influenza virus associated with human infection in China (2015). Virology 537:31-35.
14. Pulit-Penaloza JA, Pappas C, Belser JA, Sun X, Brock N, Zeng H, Tumpey TM, Maines TR. 2018. Comparative In Vitro and In Vivo Analysis of H1N1 and H1N2 Variant Influenza Viruses Isolated from Humans between 2011 and 2016. J Virol 92.
15. Pappas C, Viswanathan K, Chandrasekaran A, Raman R, Katz JM, Sasisekharan R, Tumpey TM. 2010. Receptor specificity and transmission of H2N2 subtype viruses isolated from the pandemic of 1957. PLoS One 5:e11158.

16. Pappas C, Yang H, Carney PJ, Pearce MB, Katz JM, Stevens J, Tumpey TM. 2015. Assessment of transmission, pathogenesis and adaptation of H2 subtype influenza viruses in ferrets. Virology 477:61-71.
17. Maines TR, Chen LM, Matsuoka Y, Chen H, Rowe T, Ortin J, Falcon A, Nguyen TH, Mai le Q, Sedyaningsih ER, Harun S, Tumpey TM, Donis RO, Cox NJ, Subbarao K, Katz JM. 2006. Lack of transmission of H5N1 avian-human reassortant influenza viruses in a ferret model. Proc Natl Acad Sci U S A 103:12121-6.
18. Weight AK, Belser JA, Tumpey TM, Chen J, Klibanov AM. 2014. Zanamivir conjugated to poly-L-glutamine is much more active against influenza viruses in mice and ferrets than the drug itself. Pharm Res 31:466-74.
19. Jackson S, Van Hoeven N, Chen LM, Maines TR, Cox NJ, Katz JM, Donis RO. 2009. Reassortment between avian H5N1 and human H3N2 influenza viruses in ferrets: a public health risk assessment. J Virol 83:8131-40.
20. Houser KV, Katz JM, Tumpey TM. 2013. Seasonal trivalent inactivated influenza vaccine does not protect against newly emerging variants of influenza A (H3N2v) virus in ferrets. J Virol 87:1261-3.
21. Houser KV, Pearce MB, Katz JM, Tumpey TM. 2013. Impact of prior seasonal H3N2 influenza vaccination or infection on protection and transmission of emerging variants of influenza A(H3N2)v virus in ferrets. J Virol 87:13480-9.
22. Belser JA, Gustin KM, Pearce MB, Maines TR, Zeng H, Pappas C, Sun X, Carney PJ, Villanueva JM, Stevens J, Katz JM, Tumpey TM. 2013. Pathogenesis and transmission of avian influenza A (H7N9) virus in ferrets and mice. Nature 501:556-9.
23. Pulit-Penaloza JA, Simpson N, Yang H, Creager HM, Jones J, Carney P, Belser JA, Yang G, Chang J, Zeng H, Thor S, Jang Y, Killian ML, Jenkins-Moore M, Janas-Martindale A, Dubovi E, Wentworth DE, Stevens J, Tumpey TM, Davis CT, Maines TR. 2017. Assessment of Molecular, Antigenic, and Pathological Features of Canine Influenza A(H3N2) Viruses That Emerged in the United States. J Infect Dis 216:S499-S507.
24. Sun X, Belser JA, Pulit-Penaloza JA, Brock N, Pappas C, Zanders N, Jang Y, Jones J, Tumpey TM, Davis CT, Maines TR. 2023. Pathogenesis and transmission assessment of three swine-origin influenza A(H3N2) viruses with zoonotic risk to humans isolated in the U.S from 2017-2020. J Infect Dis doi:10.1093/infdis/jiad359.
25. Pearce MB, Jayaraman A, Pappas C, Belser JA, Zeng H, Gustin KM, Maines TR, Sun X, Raman R, Cox NJ, Sasisekharan R, Katz JM, Tumpey TM. 2012. Pathogenesis and transmission of swine origin A(H3N2)v influenza viruses in ferrets. Proc Natl Acad Sci U S A 109:3944-9.
26. Sun X, Pulit-Penaloza JA, Belser JA, Pappas C, Pearce MB, Brock N, Zeng H, Creager HM, Zanders N, Jang Y, Tumpey TM, Davis CT, Maines TR. 2018. Pathogenesis and Transmission of Genetically Diverse Swine-Origin H3N2 Variant Influenza A Viruses from Multiple Lineages Isolated in the United States, 2011-2016. J Virol 92.
27. Maines TR, Lu XH, Erb SM, Edwards L, Guarner J, Greer PW, Nguyen DC, Szretter KJ, Chen LM, Thawatsupha P, Chittaganpitch M, Waicharoen S, Nguyen DT, Nguyen T, Nguyen HH, Kim JH, Hoang LT, Kang C, Phuong LS, Lim W, Zaki S, Donis RO, Cox NJ, Katz JM, Tumpey TM. 2005. Avian influenza (H5N1) viruses isolated from humans in Asia in 2004 exhibit increased virulence in mammals. J Virol 79:11788-800.
28. Nguyen DC, Uyeki TM, Jadhao S, Maines T, Shaw M, Matsuoka Y, Smith C, Rowe T, Lu X, Hall H, Xu X, Balish A, Klimov A, Tumpey TM, Swayne DE, Huynh LP, Nghiem HK, Nguyen HH, Hoang LT, Cox NJ, Katz JM. 2005. Isolation and characterization of avian influenza viruses, including highly pathogenic H5N1, from poultry in live bird markets in Hanoi, Vietnam, in 2001. J Virol 79:4201-12.
29. Pushko P, Tretyakova I, Hidajat R, Sun X, Belser JA, Tumpey TM. 2018. Multi-clade H5N1 virus-like particles: Immunogenicity and protection against H5N1 virus and effects of beta-propiolactone. Vaccine 36:4346-4353.
30. Tretyakova I, Pearce MB, Florese R, Tumpey TM, Pushko P. 2013. Intranasal vaccination with H5, H7 and H9 hemagglutinins co-localized in a virus-like particle protects ferrets from multiple avian influenza viruses. Virology 442:67-73.

31. Rao SS, Kong WP, Wei CJ, Van Hoeven N, Gorres JP, Nason M, Andersen H, Tumpey TM, Nabel GJ. 2010. Comparative efficacy of hemagglutinin, nucleoprotein, and matrix 2 protein gene-based vaccination against H5N1 influenza in mouse and ferret. PLoS One 5:e9812.
32. Smith GE, Sun X, Bai Y, Liu YV, Massare MJ, Pearce MB, Belser JA, Maines TR, Creager HM, Glenn GM, Flyer D, Pushko P, Levine MZ, Tumpey TM. 2017. Neuraminidase-based recombinant virus-like particles protect against lethal avian influenza A(H5N1) virus infection in ferrets. Virology 509:90-97.
33. Pearce MB, Pappas C, Gustin KM, Davis CT, Pantin-Jackwood MJ, Swayne DE, Maines TR, Belser JA, Tumpey TM. 2017. Enhanced virulence of clade 2.3.2.1 highly pathogenic avian influenza A H5N1 viruses in ferrets. Virology 502:114-122.
34. Thor SW, Nguyen H, Balish A, Hoang AN, Gustin KM, Nhung PT, Jones J, Thu NN, Davis W, Ngoc TN, Jang Y, Sleeman K, Villanueva J, Kile J, Gubareva LV, Lindstrom S, Tumpey TM, Davis CT, Long NT. 2015. Detection and Characterization of Clade 1 Reassortant H5N1 Viruses Isolated from Human Cases in Vietnam during 2013. PLoS One 10:e0133867.
35. Pulit-Penaloza JA, Belser JA, Brock N, Thakur PB, Tumpey TM, Maines TR. 2022. Pathogenesis and Transmissibility of North American Highly Pathogenic Avian Influenza A(H5N1) Virus in Ferrets. Emerg Infect Dis 28:1913-1915.
36. Pulit-Penaloza JA, Sun X, Creager HM, Zeng H, Belser JA, Maines TR, Tumpey TM. 2015. Pathogenesis and Transmission of Novel Highly Pathogenic Avian Influenza H5N2 and H5N8 Viruses in Ferrets and Mice. J Virol 89:10286-93.
37. Pulit-Penaloza JA, Brock N, Pappas C, Sun X, Belser JA, Zeng H, Tumpey TM, Maines TR. 2020. Characterization of highly pathogenic avian influenza H5Nx viruses in the ferret model. Sci Rep 10:12700.
38. Belser JA, Sun X, Brock N, Pulit-Penaloza JA, Jones J, Zanders N, Davis CT, Tumpey TM, Maines TR. 2020. Mammalian pathogenicity and transmissibility of low pathogenic avian influenza H7N1 and H7N3 viruses isolated from North America in 2018. Emerg Microbes Infect 9:1037-1045.
39. Belser JA, Lu X, Maines TR, Smith C, Li Y, Donis RO, Katz JM, Tumpey TM. 2007. Pathogenesis of avian influenza (H7) virus infection in mice and ferrets: enhanced virulence of Eurasian H7N7 viruses isolated from humans. J Virol 81:11139-47.
40. Belser JA, Blixt O, Chen LM, Pappas C, Maines TR, Van Hoeven N, Donis R, Busch J, McBride R, Paulson JC, Katz JM, Tumpey TM. 2008. Contemporary North American influenza H7 viruses possess human receptor specificity: Implications for virus transmissibility. Proc Natl Acad Sci U S A 105:7558-63.
41. Belser JA, Pulit-Penaloza JA, Sun X, Brock N, Pappas C, Creager HM, Zeng H, Tumpey TM, Maines TR. 2017. A Novel A(H7N2) Influenza Virus Isolated from a Veterinarian Caring for Cats in a New York City Animal Shelter Causes Mild Disease and Transmits Poorly in the Ferret Model. J Virol 91.
42. Belser JA, Davis CT, Balish A, Edwards LE, Zeng H, Maines TR, Gustin KM, Martinez IL, Fasce R, Cox NJ, Katz JM, Tumpey TM. 2013. Pathogenesis, transmissibility, and ocular tropism of a highly pathogenic avian influenza A (H7N3) virus associated with human conjunctivitis. J Virol 87:5746-54.
43. Belser JA, Creager HM, Zeng H, Maines TR, Tumpey TM. 2017. Pathogenesis, Transmissibility, and Tropism of a Highly Pathogenic Avian Influenza A(H7N7) Virus Associated With Human Conjunctivitis in Italy, 2013. J Infect Dis 216:S508-S511.
44. Sun X, Belser JA, Pulit-Penaloza JA, Zeng H, Lewis A, Shieh WJ, Tumpey TM, Maines TR. 2016. Pathogenesis and Transmission Assessments of Two H7N8 Influenza A Viruses Recently Isolated from Turkey Farms in Indiana Using Mouse and Ferret Models. J Virol 90:10936-10944.
45. Belser JA, Creager HM, Sun X, Gustin KM, Jones T, Shieh WJ, Maines TR, Tumpey TM. 2016. Mammalian Pathogenesis and Transmission of H7N9 Influenza Viruses from Three Waves, 2013-2015. J Virol 90:4647-4657.
46. Sun X, Belser JA, Pappas C, Pulit-Penaloza JA, Brock N, Zeng H, Creager HM, Le S, Wilson M, Lewis A, Stark TJ, Shieh WJ, Barnes J, Tumpey TM, Maines TR. 2019. Risk Assessment of Fifth-Wave H7N9 Influenza A Viruses in Mammalian Models. J Virol 93.

47. Belser JA, Brock N, Sun X, Jones J, Zanders N, Hodges E, Pulit-Penaloza JA, Wentworth D, Tumpey TM, Davis T, Maines TR. 2018. Mammalian Pathogenesis and Transmission of Avian Influenza A(H7N9) Viruses, Tennessee, USA, 2017. Emerg Infect Dis 24:149-152.
48. Belser JA, Sun X, Brock N, Pappas C, Pulit-Penaloza JA, Zeng H, Jang Y, Jones J, Carney PJ, Chang J, Long NV, Diep NT, Thor S, Di H, Yang G, Cook PW, Creager HM, Wang D, McFarland J, Dong PV, Wentworth DE, Tumpey TM, Barnes JR, Stevens J, Davis CT, Maines TR. 2020. Genetically and Antigenically Divergent Influenza A(H9N2) Viruses Exhibit Differential Replication and Transmission Phenotypes in Mammalian Models. J Virol 94.
49. Yang H, Chen LM, Carney PJ, Donis RO, Stevens J. 2010. Structures of receptor complexes of a North American H7N2 influenza hemagglutinin with a loop deletion in the receptor binding site. PLoS Pathog 6:e1001081.
