# Supplementary material for: Machine learning approaches for influenza A virus risk assessment identifies predictive correlates using ferret model in vivo data
Source: Commun Biol. 2024 Aug 1;7:927. doi: 10.1038/s42003-024-06629-0 (PMC11294530; doi:10.1038/s42003-024-06629-0)
Supplement: Supplementary file 2 — Description of Additional Supplementary Files [file 42003_2024_6629_MOESM2_ESM.docx]

**Description of Additional Supplementary Files**

File: Supplementary Data 1.

Description: Table of features tested in each iteration of model testing. Includes the classification model (lethality, morbidity, transmission), the type of dataset used for testing (standard, combined, molecular), the outcome being classified. Descriptors of each feature are provided below.

File: Supplementary Data 2.

Description: Table of all 14-evaluation metrics, for all 11 machine learning algorithms tested, for each iterative test of lethality classification models on the internal standard dataset.

File: Supplementary Data 3

Description: Table of all 14-evaluation metrics, for all 11 machine learning algorithms tested, for each iterative test of lethality classification models on the internal molecular only and combined datasets.

File: Supplementary Data 4

Description: Table of all 14-evaluation metrics, for all 11 machine learning algorithms tested, for each iterative test of morbidity classification models on the internal standard dataset.

File: Supplementary Data 5

Description: Table of all 14 evaluation metrics, for all 11 machine learning algorithms tested, for each iterative test of morbidity classification models on the internal molecular only and combined datasets.

File: Supplementary Data 6

Description: Table of all 14 evaluation metrics, for all 11 machine learning algorithms tested, for each iterative test of transmission classification models on the internal standard dataset.

File: Supplementary Data 7

Description: Table of all 14 evaluation metrics, for all 11 machine learning algorithms tested, for each iterative test of transmission classification models on the internal molecular only and combined datasets.

File: Supplementary Data 8

Description: Table of final model parameters used for each top model showing the machine learning algorithm, and the relevant parameters that were tuned with the final value for each.

File: Supplementary Data 9

Description: Table of feature importance for each iterative test of lethality classification models on the internal standard dataset. The top importance value is scaled to 100 and remaining features are relatively ranked accordingly.

File: Supplementary Data 10

Description: Table of feature importance for each iterative test of lethality classification models on the internal molecular only and combined datasets. The top importance value is scaled to 100 and remaining features are relatively ranked accordingly.

File: Supplementary Data 11.

Description: Table of feature importance for each iterative test of morbidity classification models on the internal standard dataset. The top importance value is scaled to 100 and remaining features are relatively ranked accordingly.

File: Supplementary Data 12

Description: Table of feature importance for each iterative test of morbidity classification models on the internal molecular only and combined datasets. The top importance value is scaled to 100 and remaining features are relatively ranked accordingly.

File: Supplementary Data 13

Description: Table of feature importance for each iterative test of transmission classification models on the internal standard dataset. The top importance value is scaled to 100 and remaining features are relatively ranked accordingly.

File: Supplementary Data 14

Description: Table of feature importance for each iterative test of transmission classification models on the internal molecular only and combined datasets. The top importance value is scaled to 100 and remaining features are relatively ranked accordingly.

File: Supplementary Data 15

Description: Table of predictive power scores for selected features against the outcome variable (y).

File: Supplementary Data 16

Description: Table of summary statistics from the internal dataset grouped by HA, MBAA, RBA-PBS combination, and lethality outcome used to create simulated dataset. The mean (m), standard deviation (sd), skew (sk), and kurtosis (kr) are shown for the features wt_loss, tem5, and AUC_6.

File: Supplementary Data 17

Description: Table of the 2,900 simulated data observations.

File: Supplementary Data 18

Description: Table of influenza A viruses from published literature as an external validation dataset to test the molecular only based models for lethality and transmission. Includes virus metadata, and source references with number of observations used for lethality and transmission classification models.
